# Socioeconomic determinants, risk perception, and food safety concerns of African Giant snail consumption in Yenagoa Metropolis, Nigeria

**DOI:** 10.3389/fpubh.2026.1865889

**Published:** 2026-09-10

**Authors:** Sylvester Chibueze Izah, Tarvie Jacob Jack, Esther Ugo Alum, Vivian Ibienebakabobo Promise, Matthew Chidozie Ogwu, Irene Nneka Ohanusi

**Affiliations:** 1 Department of Community Medicine, Faculty of Clinical Sciences, Bayelsa Medical University, Yenagoa, Bayelsa, Nigeria; 2 Department of Microbiology, Faculty of Science, Bayelsa Medical University, Yenagoa, Bayelsa, Nigeria; 3 Department of Medical Social Work, Faculty of Health Sciences, Bayelsa Medical University, Yenagoa, Bayelsa, Nigeria; 4 Department of Research and Publications, Kampala International University, Kampala International University, Kampala, Uganda; 5 Department of Biochemistry, Faculty of Science, Ebonyi State University, Abakaliki, Ebonyi, Nigeria; 6 Department of Public Health, Faculty of Health Sciences, Bayelsa Medical University, Yenagoa, Bayelsa, Nigeria; 7 Goodnight Family Department of Sustainable Development, Appalachian State University, Boone, NC, United States; 8 Department of Microbiology, Faculty of Natural Sciences, Hezekiah University, Umudi, Nkwerre, Nigeria

**Keywords:** Bonferroni correction, consumer behaviour, food safety, risk perception, snails, trace metals, Yenagoa

## Abstract

**Background:**

Environmental contamination in the Niger Delta has generated concern about the safety of foods harvested from natural environments. Laboratory studies have shown that African giant snails can accumulate trace elements.

**Objective:**

To assess socioeconomic correlates of African giant snail consumption, awareness and food-safety perceptions, perceived public-health implications, and support for community and policy measures among adult respondents in Yenagoa Metropolis, Nigeria.

**Methods:**

A descriptive cross-sectional survey was conducted among 391 adults recruited through a multistage community- and venue-based procedure. A 40-item questionnaire comprised four ten-item domains: (1) socioeconomic factors related to snail consumption; (2) awareness, perceptions, and coping strategies concerning snail safety; (3) perceived public-health implications and vulnerability; and (4) community-based interventions and policy measures, each scored on a five-point response scale. Domain scores were analysed as mean composite scores. Descriptive statistics, Pearson chi-square tests, Bonferroni-adjusted item-level significance tests, Cramér’s V, multiple linear regression with 95% confidence intervals, and a secondary repeated-measures general linear model with Bonferroni-adjusted pairwise comparisons were used.

**Results:**

Respondents were predominantly female (59.3%), students (53.2%), and highly educated (53.5% tertiary; 34.3% postgraduate. In the socioeconomic-item analyses, education was associated with several consumption-related items after Bonferroni correction, while income was not associated with the relevant item (*χ*^2^ = 15.695, df = 16, raw *p* = 0.474, Bonferroni-adjusted *p* = 1.000). In occupation-based awareness analyses, associations that remained after correction included awareness of environmental pollution, checking snail sources, and trust in vendor hygiene. The regression models explained 14.0% of variance in the socioeconomic-consumption composite, 12.0% in awareness, 23.6% in perceived public-health implications, and 19.9% in policy support. Marital status (B = 0.258, 95% CI 0.121 to 0.396) and education (B = -0.272, 95% CI -0.368 to −0.176) were independently associated with the socioeconomic-consumption composite. The socioeconomic-consumption composite was associated with awareness (B = 0.370, 95% CI 0.250 to 0.489); both socioeconomic-consumption and awareness were associated with perceived public-health implications; and age, education, and perceived public-health implications were associated with policy support.

**Conclusion:**

The findings indicate an awareness–practice gap and substantial public support for food-safety interventions. However, these findings reflect consumer perceptions and reported behaviours rather than measured trace-metal contamination or direct exposure among respondents.

## 1 Introduction

Environmental pollution is a major public-health and ecological concern, particularly where industrial activity, urbanisation, and weak waste-management systems increase the movement of contaminants through soil, water, air, and food chains (1). In Nigeria, trace metals including lead, cadmium, chromium, and nickel have been reported in environmental media and foods; their persistence and potential for bioaccumulation make dietary exposure an important subject of environmental-health research (2–5).

Bayelsa State is located in the Niger Delta, where petroleum activities, urban expansion, waste disposal, and other anthropogenic pressures have raised environmental-quality concerns. Studies in Bayelsa have reported trace elements and other pollutants in water, air, sediment, fish, and related environmental matrices (2, 3, 6–12). These findings provide environmental context. However, trace metals have been reported in several food materials and some food commonly consumed in Yenagoa metropolis have not been studied with regard to trace metals have not been studied.

African giant snails are widely consumed in southern Nigeria and may be harvested from natural environments where soil, vegetation, and decomposing organic matter form part of their ecological contact and feeding conditions. Laboratory investigations in Nigeria have reported accumulation of trace metals in edible land snails, although concentrations vary substantially by species and location (13–15).

Evidence on possible health effects in this manuscript is drawn from prior toxicological and food-contamination studies, not from biological or environmental measurements made in the present survey. Previous Nigerian studies have assessed estimated dietary intake, non-carcinogenic hazard, or carcinogenic risk from chemically analysed snail samples (14–16). Those findings establish the plausibility of food-safety concern, but they do not demonstrate that the respondents in the present study were exposed to hazardous concentrations.

Chronic exposure to elevated concentrations of some trace metals can adversely affect human health, depending on dose, duration, chemical form, and susceptibility (17). Nigerian food and environmental studies have therefore used chemical analysis and risk-assessment models to evaluate potential dietary hazards. Such toxicological evidence is background for the present survey and should not be confused with measured exposure among its respondents (14, 18).

Accordingly, a consumer-focused study can complement laboratory evidence by examining whether residents know about potential contamination pathways, how they select and handle snails, which population groups they perceive as vulnerable, and which interventions they support. Such perceptions are important for risk communication and programme design, but they should not be treated as direct measurements of contamination, dose, or health effect.

Existing studies in Nigeria have largely focused on laboratory-based assessments of heavy metal concentrations in snails (19), with limited attention to consumer awareness, perceptions, and behavioural responses. Furthermore, previous research indicates that contamination levels vary significantly across locations and species, suggesting that environmental context plays a critical role in determining exposure risk (13, 14).

Available studies show that trace-metal concentrations in edible snails are not uniform and can vary with species, harvesting location, and local environmental conditions. Investigations in southern Nigeria have reported elevated concentrations in some industrial, waste-affected, or otherwise impacted settings, whereas results from other settings are lower, reinforcing the need for location-specific chemical assessment rather than general assumptions about snail safety (13, 15, 20–24).

Conversely, snails harvested from relatively unpolluted rural ecosystems may contain metal concentrations within acceptable safety limits, suggesting that contamination risk is context-dependent rather than universal. This variability stresses the importance of environmental monitoring, traceability of harvesting sources, and context-specific food safety assessments rather than generalised assumptions about snail safety. Despite growing evidence on environmental contamination, there remains limited integration between laboratory-based toxicological studies and consumer-focused behavioural research. In particular, little is known about how consumers perceive contamination risks, verify food sources, and respond to food safety information in high-risk environments such as the Niger Delta.

Therefore, this study assessed socioeconomic correlates of African giant snail consumption, awareness and food-safety perceptions, perceived public-health implications, and policy preferences among adult respondents in Yenagoa Metropolis. The findings are intended to inform risk-communication priorities, identify areas of public support for food-safety action, and generate hypotheses for future environmental and toxicological investigation.

## 2 Methodology

### 2.1 Study area

The study was conducted in Yenagoa Metropolis, Bayelsa State, within the Niger Delta region of Nigeria (approximately 4.92°N, 6.26°E). The metropolis contains multiple urban and peri-urban communities and has experienced rapid population growth, petroleum-related activity, commercial expansion, and environmental pressures. Studies from Bayelsa State have documented trace elements and other pollutants in surface water, sediment, atmospheric particulate matter, fish, and other environmental matrices (2, 3, 8–12). These prior findings provided the environmental-health context for studying consumer perceptions; they were not treated as measurements of contamination in the snails consumed by participants in this survey.

### 2.2 Study design

A descriptive cross-sectional design was used to collect questionnaire data at one period in time. This design permits estimation of response distributions and assessment of statistical associations but does not establish temporal sequence or causality. Relevant STROBE guidance for cross-sectional observational studies informed reporting (25).

### 2.3 Study population

The target population comprised adults aged 18 years or older who had lived in Yenagoa Metropolis for at least 6 months and who reported prior knowledge of or experience with African giant snails.

### 2.4 Inclusion and exclusion criteria

Eligible participants were adults aged 18 years or older, resident in Yenagoa for at least 6 months, and familiar with or experienced in African giant snail consumption. Visitors, persons younger than 18 years, and persons unfamiliar with snail consumption were excluded.

### 2.5 Sample size determination

The sample size was determined using Cochran’s formula as described by Ogbeibu (26):

$$n=[Z^{2}\times p(1-p)]/d^{2}$$

Where:

-
n = required sample size
-
Z = standard normal deviate at 95% confidence level (1.96)
-
p = estimated population proportion (0.5)
-
d = margin of error (0.05)

Substitution yielded a minimum sample size of 384 respondents. To account for non-response and incomplete data, an additional 10% was included:

n_adjusted=384+(0.10×384)=422

A total of 422 questionnaires were distributed, of which 391 were correctly completed and analyzed, representing a response rate of 92.7%. This final sample size was considered adequate for statistical analysis.

### 2.6 Sampling technique

A multistage sampling technique was employed to obtain respondents from different parts of Yenagoa Metropolis and to enhance the geographical and socioeconomic coverage of the study population. The sampling procedure involved the selection of communities, data-collection locations within the selected communities, and eligible respondents at the identified locations. In the first stage, the communities comprising the metropolis, including Igbogene, Yenigue, Akenfa, Agudama, Akempai, Edepie, Etegwe, Okutukutu, Opolo, Biogbolo, Yenizuegene, Kpansia, Yenizue-Epie, Okaka, Ekeki, Amarata, Onopa, Ovom, Swali, and Azikoro (27), were listed. In the second stage, 8 out of the 20 communities were selected from the community sampling frame using simple random sampling without replacement. In the third stage, specific data-collection points were chosen to provide access to adults from different occupational, educational, social, and residential backgrounds. The data-collection points included markets, residential areas, food joints and other commercial locations, as well as public and social institutions such as universities, government ministries, and offices. In the fourth and final stage, eligible respondents were recruited from the selected data-collection points according to the inclusion criteria of the study. Where a complete sampling frame of eligible respondents was prepared, each eligible individual was assigned an identification number, after which respondents were selected randomly using simple balloting. Respondents who did not satisfy the eligibility criteria or who declined to participate were excluded.

### 2.7 Data collection instrument

The structured instrument contained a demographic section followed by 40 five-point items organised *a priori* into four ten-item domains: (1) socioeconomic factors related to snail consumption; (2) awareness, perceptions, and coping strategies concerning snail safety; (3) perceived public-health implications and vulnerability; and (4) community-based interventions and policy measures. Response anchors were ordered from lower to higher endorsement, frequency, concern, or perceived truth depending on the item wording.

For regression analyses, each domain score was specified as the arithmetic mean of its 10 constituent items: (1) socioeconomic factors related to snail consumption; (2) awareness, perceptions, and coping strategies concerning snail safety; (3) perceived public-health implications and vulnerability; and (4) community-based interventions and policy measures. Higher values indicated greater average endorsement or intensity within the relevant domain. Items were oriented from lower to higher endorsement, frequency, concern, or perceived truth; no reverse-scored item was done. These four prespecified mean scores were used as composite outcomes or predictors in the regression sequence. Item-level descriptive frequencies were retained to show the distribution of responses, while composite means were used only for multivariable regression and the secondary within-respondent comparison of domain means.

### 2.8 Validity and reliability of the instrument

Content validity was assessed through expert review of item relevance, clarity, and coverage of food-safety, environmental-health, and consumer-behaviour content. The questionnaire was pretested with 20 adults in Amassoma, outside the main Yenagoa study area, to assess wording, comprehension, administration time, and internal consistency. The 20 adults were selected based on simple random sampling techniques. The pretest internal-consistency coefficients ranged from 0.72 to 0.88 across sections of the instruments. These pretested values are distinguished from the reliability coefficients calculated in the main study sample as shown in the results sections.

### 2.9 Data collection procedure

Data collection was conducted at the selected community venues at different times of the day. Trained data collectors explained the study purpose, eligibility requirements, voluntary nature of participation, and confidentiality protections before questionnaire administration. Informed consent was obtained from every participant before participation.

### 2.10 Data analysis

Data were analysed in IBM SPSS Statistics version 27. Frequencies and percentages summarised categorical and item-level responses; means and standard deviations summarised the four composite domain scores. In the stratified crosstabulation tables, each response cell is reported as frequency with the corresponding within-stratum row percentage, while the Total n (%) column gives the stratum total and its percentage of valid responses for that questionnaire item. Pearson chi-square tests assessed associations between education or occupation and each questionnaire item. Because 10 chi-square hypotheses were tested within each demographic-by-domain family, family-wise error was controlled with a Bonferroni adjustment: p_adj = min(10 × p_raw,1.000), equivalent to an item-level threshold of 0.005 for family-wise α = 0.05. Raw and adjusted *p*-values are reported with Cramér’s V as an effect-size measure. Where SPSS displayed *p* = 0.000, the raw *p*-value was approximated from the reported Pearson χ^2^ statistic and degrees of freedom using the chi-square survival function.

Four sequential multiple linear regression models were fitted to the prespecified mean domain scores. Model 1 regressed socioeconomic factors related to snail consumption on age, sex, marital status, education, and occupation. Model 2 regressed awareness, perceptions, and coping strategies concerning snail safety on those variables plus the Domain 1 score. Model 3 regressed perceived public-health implications and vulnerability on the Model 2 predictors plus the Domain 2 score. Model 4 regressed community-based interventions and policy measures on the Model 3 predictors plus the Domain 3 score. Unstandardized B, standard error, standardized β, t, p, 95% confidence intervals, tolerance, and variance inflation factor (VIF) are reported. VIF values ranged from approximately 1.05 to 1.98, providing no indication of serious multicollinearity. Durbin–Watson statistics ranged from 1.577 to 1.952. Casewise and influence diagnostics showed few standardized residuals beyond ±3 and no Cook’s distance approaching 1.

As a secondary descriptive analysis, the four 1–5 domain means were compared within respondents using a repeated-measures general linear model. Mauchly’s test indicated departure from sphericity (W = 0.901, χ^2^ = 40.587, df = 5, *p* < 0.001); therefore, the Greenhouse–Geisser correction (ε = 0.932) was used for the omnibus within-subject test. Pairwise domain comparisons used SPSS estimated marginal means with Bonferroni adjustment. This domain-level Bonferroni procedure is separate from the item-level Bonferroni correction applied to the chi-square families.

### 2.11 Ethical considerations

Ethical approval was obtained from the Bayelsa Medical University Research and Ethics Committee. Participation was voluntary, informed consent was obtained before questionnaire administration, no personal identifiers were collected, and respondents were assured of confidentiality and anonymity. Data were used strictly for research purposes.

## 3 Results and discussion

### 3.1 Results

#### 3.1.1 Socioeconomic and demographic characteristics of respondents

Table 1 provides the complete socioeconomic and demographic distribution of the achieved sample, including age, sex, marital status, education, occupation, and religion. The analysis included 391 respondents. The achieved sample was disproportionately composed of students and persons with higher education; therefore, the demographic distribution is presented as the profile of the achieved sample rather than the population structure of Yenagoa Metropolis.

**Table 1:** Socioeconomic and demographic characteristics of respondents (*n* = 391).

Variable	Category	Frequency (n)	Percentage (%)
Age	18–25	172	44.0
	>25	219	56.0
Sex	Male	159	40.7
	Female	232	59.3
Marital Status	Single	243	62.1
	Married	132	33.8
	Divorced	12	3.1
	Widowed	3	0.8
	Separated	1	0.3
Education	No formal education	2	0.5
	Primary	6	1.5
	Secondary	40	10.2
	Tertiary	209	53.5
	Postgraduate	134	34.3
Occupation	Student	208	53.2
	Trader	27	6.9
	Civil servant	109	27.9
	Artisan	20	5.1
	Unemployed	18	4.6
	Others	9	2.3
Religion	Christianity	373	95.4
	Islam	5	1.3
	Others	13	3.3

Values are expressed as frequency (n) and percentage (%).

Using mutually exclusive labels for reporting, 44.0% were aged 18–25 years and 56.0% were older than 25 years. Females constituted 59.3% of the sample. Most respondents were single (62.1%), and 87.8% had tertiary (53.5%) or postgraduate (34.3%) education. Students formed 53.2% of respondents, followed by civil servants (27.9%), traders (6.9%), artisans (5.1%), unemployed respondents (4.6%), and other occupations (2.3%).

**Figure 1:**
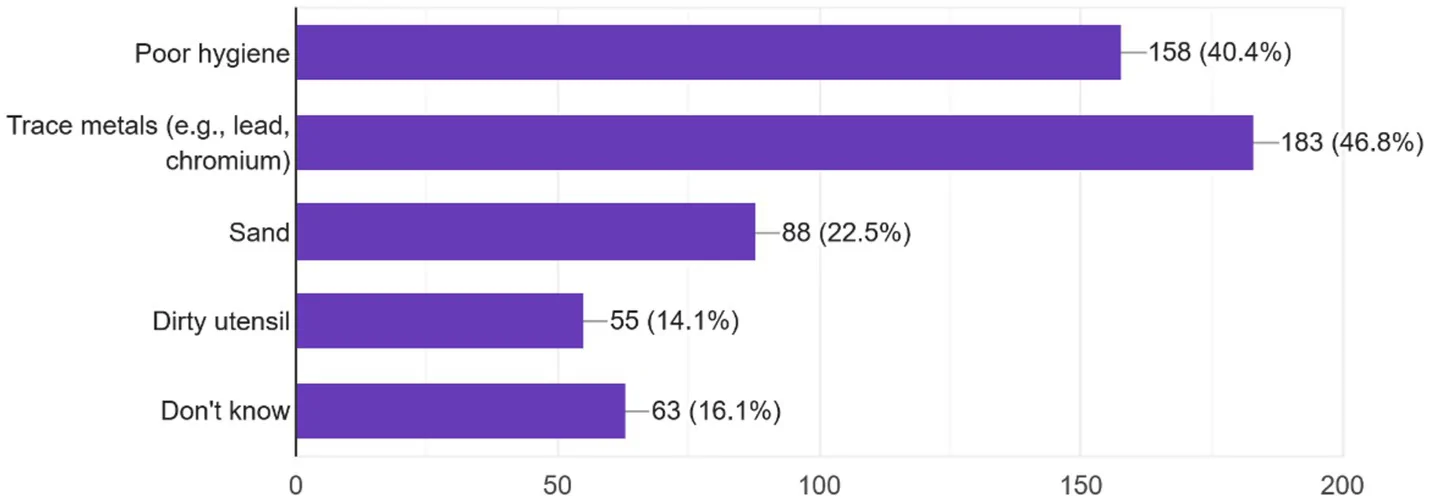
Perceived sources of contamination of african giant snails among respondents in Yenagoa Metropolis, Nigeria (multiple responses allowed).

Figure 1 summarises respondents’ multiple responses concerning perceived sources of contamination of African giant snails. Trace metals were selected by 183 respondents (46.8%), poor hygiene by 158 (40.4%), sand by 88 (22.5%), dirty utensils by 55 (14.1%), and 63 respondents (16.1%) reported that they did not know a contamination source. These values describe perceived contamination pathways only.

#### 3.1.2 Socioeconomic factors related to snail consumption

Tables 2, 3 present the education- and occupation-based crosstabulations for the 10 items in the socioeconomic factors related to snail consumption domain. After Bonferroni correction within the 10 education-based socioeconomic tests, significant associations remained for perceived nutritional benefits (adjusted *p* = 0.0039), perception of snails as a cheaper protein source (adjusted *p* < 0.001), gender-related preference (adjusted *p* < 0.001), and the perception that snails are more commonly consumed in low-income households (adjusted *p* = 0.0009). The cross-tabulation in Table 2 shows the clearest contrasts for the latter three items. For the cheaper-protein statement, 150/209 (71.8%) tertiary and 103/134 (76.9%) postgraduate respondents disagreed or strongly disagreed, whereas 17/40 (42.5%) secondary respondents agreed or strongly agreed. For gender-related preference, 176/209 (84.2%) tertiary and 113/134 (84.3%) postgraduate respondents disagreed or strongly disagreed, compared with 19/40 (47.5%) secondary respondents who agreed or strongly agreed. For the low-income-household statement, 21/40 (52.5%) secondary respondents agreed or strongly agreed, compared with 35/209 (16.7%) tertiary and 17/134 (12.7%) postgraduate respondents. The income item was not associated with education (*χ*^2^ = 15.695, df = 16, raw *p* = 0.4744, adjusted *p* = 1.0000).

**Table 2:** Education-based association for socioeconomic factors related to snail consumption in Yenagoa Metropolis, Nigeria.

Questionnaire item	Educational level	Strongly disagree *n* (%)	Disagree *n* (%)	Neutral *n* (%)	Agree *n* (%)	Strongly agree n (%)	Total *n* (%)	*χ*^2^ (df)	Raw *p*	Bonferroni- adjusted p	Cramér’s V
My income level influences how frequently I purchase African giant snails.	No formal education	1 (50.0)	0 (0.0)	0 (0.0)	1 (50.0)	0 (0.0)	2 (0.5)	15.695 (16)	0.4744	1.0000	0.100
	Primary	1 (16.7)	0 (0.0)	2 (33.3)	3 (50.0)	0 (0.0)	6 (1.5)				
	Secondary	2 (5.0)	5 (12.5)	8 (20.0)	15 (37.5)	10 (25.0)	40 (10.2)				
	Tertiary	24 (11.5)	43 (20.6)	40 (19.1)	66 (31.6)	36 (17.2)	209 (53.5)				
	Postgraduate	25 (18.7)	24 (17.9)	24 (17.9)	40 (29.9)	21 (15.7)	134 (34.3)				
Educational background affects my understanding of the nutritional benefits of snails.	No formal education	0 (0.0)	1 (50.0)	0 (0.0)	1 (50.0)	0 (0.0)	2 (0.5)	42.069 (16)	0.0004	0.0039	0.164
	Primary	1 (16.7)	0 (0.0)	4 (66.7)	1 (16.7)	0 (0.0)	6 (1.5)				
	Secondary	2 (5.0)	6 (15.0)	14 (35.0)	10 (25.0)	8 (20.0)	40 (10.2)				
	Tertiary	30 (14.4)	57 (27.3)	40 (19.1)	63 (30.1)	19 (9.1)	209 (53.5)				
	Postgraduate	38 (28.4)	38 (28.4)	16 (11.9)	29 (21.6)	13 (9.7)	134 (34.3)				
Employment status affects my ability to afford snails regularly.	No formal education	1 (50.0)	0 (0.0)	0 (0.0)	1 (50.0)	0 (0.0)	2 (0.5)	29.669 (16)	0.0198	0.1980	0.138
	Primary	1 (16.7)	1 (16.7)	3 (50.0)	1 (16.7)	0 (0.0)	6 (1.5)				
	Secondary	1 (2.5)	7 (17.5)	13 (32.5)	12 (30.0)	7 (17.5)	40 (10.2)				
	Tertiary	33 (15.8)	56 (26.8)	44 (21.1)	61 (29.2)	15 (7.2)	209 (53.5)				
	Postgraduate	32 (23.9)	42 (31.3)	17 (12.7)	31 (23.1)	12 (9.0)	134 (34.3)				
I consume snails more often because they are readily available in my neighborhood.	No formal education	0 (0.0)	2 (100.0)	0 (0.0)	0 (0.0)	0 (0.0)	2 (0.5)	26.764 (16)	0.0442	0.4418	0.131
	Primary	1 (16.7)	2 (33.3)	3 (50.0)	0 (0.0)	0 (0.0)	6 (1.5)				
	Secondary	2 (5.0)	11 (27.5)	9 (22.5)	10 (25.0)	8 (20.0)	40 (10.2)				
	Tertiary	33 (15.8)	76 (36.4)	46 (22.0)	43 (20.6)	11 (5.3)	209 (53.5)				
	Postgraduate	24 (17.9)	51 (38.1)	25 (18.7)	29 (21.6)	5 (3.7)	134 (34.3)				
Members of my household eat snails regularly due to cultural food preferences.	No formal education	0 (0.0)	1 (50.0)	1 (50.0)	0 (0.0)	0 (0.0)	2 (0.5)	30.934 (16)	0.0137	0.1372	0.141
	Primary	1 (16.7)	1 (16.7)	4 (66.7)	0 (0.0)	0 (0.0)	6 (1.5)				
	Secondary	1 (2.5)	11 (27.5)	8 (20.0)	13 (32.5)	7 (17.5)	40 (10.2)				
	Tertiary	31 (14.8)	85 (40.7)	41 (19.6)	45 (21.5)	7 (3.3)	209 (53.5)				
	Postgraduate	18 (13.4)	55 (41.0)	25 (18.7)	29 (21.6)	7 (5.2)	134 (34.3)				
I am more likely to consume snails because they are cheaper than other protein sources.	No formal education	2 (100.0)	0 (0.0)	0 (0.0)	0 (0.0)	0 (0.0)	2 (0.5)	55.144 (16)	<0.0001	<0.0001	0.188
	Primary	1 (16.7)	1 (16.7)	2 (33.3)	1 (16.7)	1 (16.7)	6 (1.5)				
	Secondary	5 (12.5)	8 (20.0)	10 (25.0)	10 (25.0)	7 (17.5)	40 (10.2)				
	Tertiary	58 (27.8)	92 (44.0)	34 (16.3)	20 (9.6)	5 (2.4)	209 (53.5)				
	Postgraduate	37 (27.6)	66 (49.3)	22 (16.4)	6 (4.5)	3 (2.2)	134 (34.3)				
My gender influences my preference for snail consumption.	No formal education	1 (50.0)	0 (0.0)	1 (50.0)	0 (0.0)	0 (0.0)	2 (0.5)	96.455 (16)	<0.0001	<0.0001	0.248
	Primary	1 (16.7)	1 (16.7)	1 (16.7)	1 (16.7)	2 (33.3)	6 (1.5)				
	Secondary	9 (22.5)	7 (17.5)	5 (12.5)	14 (35.0)	5 (12.5)	40 (10.2)				
	Tertiary	89 (42.6)	87 (41.6)	21 (10.0)	7 (3.3)	5 (2.4)	209 (53.5)				
	Postgraduate	50 (37.3)	63 (47.0)	14 (10.4)	3 (2.2)	4 (3.0)	134 (34.3)				
Snails are more commonly consumed in low-income households in this area.	No formal education	1 (50.0)	0 (0.0)	1 (50.0)	0 (0.0)	0 (0.0)	2 (0.5)	46.280 (16)	<0.0001	0.0009	0.172
	Primary	1 (16.7)	0 (0.0)	2 (33.3)	2 (33.3)	1 (16.7)	6 (1.5)				
	Secondary	7 (17.5)	9 (22.5)	3 (7.5)	15 (37.5)	6 (15.0)	40 (10.2)				
	Tertiary	52 (24.9)	91 (43.5)	31 (14.8)	28 (13.4)	7 (3.3)	209 (53.5)				
	Postgraduate	39 (29.1)	62 (46.3)	16 (11.9)	12 (9.0)	5 (3.7)	134 (34.3)				
I first learned about snail consumption from family traditions.	No formal education	0 (0.0)	0 (0.0)	1 (50.0)	1 (50.0)	0 (0.0)	2 (0.5)	30.514 (16)	0.0155	0.1551	0.140
	Primary	1 (16.7)	0 (0.0)	3 (50.0)	0 (0.0)	2 (33.3)	6 (1.5)				
	Secondary	3 (7.5)	8 (20.0)	5 (12.5)	16 (40.0)	8 (20.0)	40 (10.2)				
	Tertiary	28 (13.4)	55 (26.3)	37 (17.7)	67 (32.1)	22 (10.5)	209 (53.5)				
	Postgraduate	25 (18.7)	22 (16.4)	11 (8.2)	55 (41.0)	21 (15.7)	134 (34.3)				
The size of my household determines how much snail we consume weekly.	No formal education	0 (0.0)	0 (0.0)	1 (50.0)	1 (50.0)	0 (0.0)	2 (0.5)	24.828 (16)	0.0729	0.7292	0.126
	Primary	1 (16.7)	2 (33.3)	1 (16.7)	1 (16.7)	1 (16.7)	6 (1.5)				
	Secondary	3 (7.5)	4 (10.0)	9 (22.5)	15 (37.5)	9 (22.5)	40 (10.2)				
	Tertiary	30 (14.4)	56 (26.8)	40 (19.1)	65 (31.1)	18 (8.6)	209 (53.5)				
	Postgraduate	28 (20.9)	35 (26.1)	16 (11.9)	47 (35.1)	8 (6.0)	134 (34.3)				

Each response entry is reported as *n* (%); Values are expressed as frequency (n) and percentage (%); Values reported in the format n/N (%) in text indicate the number of respondents with the specified response (*n*), the total respondents in the relevant subgroup (*N*), and the corresponding percentage calculated as n divided by N multiplied by 100; Pearson *χ*^2^ with degrees of freedom, raw p-values, Bonferroni-adjusted *p*-values, and Cramér’s V are reported at item level. Bonferroni adjustment controls family-wise *α* = 0.05 across the 10 tests in each demographic-by-domain family.

**Table 3:** Occupation-based association for socioeconomic factors related to snail consumption in Yenagoa Metropolis, Nigeria.

Questionnaire item	Occupation	Strongly disagree n (%)	Disagree *n* (%)	Neutral *n* (%)	Agree *n* (%)	Strongly agree n (%)	Total *n* (%)	*χ*^2^ (df)	Raw *p*	Bonferroni- adjusted p	Cramér’s V
My income level influences how frequently I purchase African giant snails.	Student	31 (14.9)	44 (21.2)	40 (19.2)	65 (31.2)	28 (13.5)	208 (53.2)	21.974 (20)	0.3419	1.0000	0.119
	Trader	0 (0.0)	5 (18.5)	5 (18.5)	12 (44.4)	5 (18.5)	27 (6.9)				
	Civil servant	15 (13.8)	17 (15.6)	18 (16.5)	34 (31.2)	25 (22.9)	109 (27.9)				
	Artisan	1 (5.0)	1 (5.0)	4 (20.0)	7 (35.0)	7 (35.0)	20 (5.1)				
	Unemployed	4 (22.2)	3 (16.7)	5 (27.8)	5 (27.8)	1 (5.6)	18 (4.6)				
	Others	2 (22.2)	2 (22.2)	2 (22.2)	2 (22.2)	1 (11.1)	9 (2.3)				
Educational background affects my understanding of the nutritional benefits of snails.	Student	33 (15.9)	51 (24.5)	44 (21.2)	64 (30.8)	16 (7.7)	208 (53.2)	45.572 (20)	0.0009	0.0092	0.171
	Trader	1 (3.7)	4 (14.8)	7 (25.9)	9 (33.3)	6 (22.2)	27 (6.9)				
	Civil servant	30 (27.5)	34 (31.2)	14 (12.8)	18 (16.5)	13 (11.9)	109 (27.9)				
	Artisan	1 (5.0)	1 (5.0)	7 (35.0)	7 (35.0)	4 (20.0)	20 (5.1)				
	Unemployed	5 (27.8)	7 (38.9)	1 (5.6)	4 (22.2)	1 (5.6)	18 (4.6)				
	Others	1 (11.1)	5 (55.6)	1 (11.1)	2 (22.2)	0 (0.0)	9 (2.3)				
Employment status affects my ability to afford snails regularly.	Student	38 (18.3)	58 (27.9)	47 (22.6)	54 (26.0)	11 (5.3)	208 (53.2)	40.618 (20)	0.0042	0.0417	0.161
	Trader	2 (7.4)	5 (18.5)	6 (22.2)	8 (29.6)	6 (22.2)	27 (6.9)				
	Civil servant	21 (19.3)	30 (27.5)	13 (11.9)	30 (27.5)	15 (13.8)	109 (27.9)				
	Artisan	1 (5.0)	1 (5.0)	8 (40.0)	8 (40.0)	2 (10.0)	20 (5.1)				
	Unemployed	5 (27.8)	6 (33.3)	2 (11.1)	5 (27.8)	0 (0.0)	18 (4.6)				
	Others	1 (11.1)	6 (66.7)	1 (11.1)	1 (11.1)	0 (0.0)	9 (2.3)				
I consume snails more often because they are readily available in my neighborhood.	Student	36 (17.3)	80 (38.5)	43 (20.7)	41 (19.7)	8 (3.8)	208 (53.2)	30.401 (20)	0.0636	0.6361	0.139
	Trader	1 (3.7)	9 (33.3)	6 (22.2)	5 (18.5)	6 (22.2)	27 (6.9)				
	Civil servant	17 (15.6)	41 (37.6)	21 (19.3)	24 (22.0)	6 (5.5)	109 (27.9)				
	Artisan	1 (5.0)	4 (20.0)	7 (35.0)	5 (25.0)	3 (15.0)	20 (5.1)				
	Unemployed	4 (22.2)	6 (33.3)	5 (27.8)	3 (16.7)	0 (0.0)	18 (4.6)				
	Others	1 (11.1)	2 (22.2)	1 (11.1)	4 (44.4)	1 (11.1)	9 (2.3)				
Members of my household eat snails regularly due to cultural food preferences.	Student	26 (12.5)	90 (43.3)	44 (21.2)	41 (19.7)	7 (3.4)	208 (53.2)	44.729 (20)	0.0012	0.0120	0.169
	Trader	0 (0.0)	7 (25.9)	6 (22.2)	8 (29.6)	6 (22.2)	27 (6.9)				
	Civil servant	18 (16.5)	43 (39.4)	20 (18.3)	25 (22.9)	3 (2.8)	109 (27.9)				
	Artisan	1 (5.0)	3 (15.0)	6 (30.0)	7 (35.0)	3 (15.0)	20 (5.1)				
	Unemployed	4 (22.2)	8 (44.4)	2 (11.1)	4 (22.2)	0 (0.0)	18 (4.6)				
	Others	2 (22.2)	2 (22.2)	1 (11.1)	2 (22.2)	2 (22.2)	9 (2.3)				
I am more likely to consume snails because they are cheaper than other protein sources.	Student	58 (27.9)	92 (44.2)	34 (16.3)	20 (9.6)	4 (1.9)	208 (53.2)	66.391 (20)	<0.0001	<0.0001	0.206
	Trader	3 (11.1)	7 (25.9)	7 (25.9)	5 (18.5)	5 (18.5)	27 (6.9)				
	Civil servant	34 (31.2)	53 (48.6)	15 (13.8)	4 (3.7)	3 (2.8)	109 (27.9)				
	Artisan	1 (5.0)	2 (10.0)	7 (35.0)	6 (30.0)	4 (20.0)	20 (5.1)				
	Unemployed	5 (27.8)	9 (50.0)	3 (16.7)	1 (5.6)	0 (0.0)	18 (4.6)				
	Others	2 (22.2)	4 (44.4)	2 (22.2)	1 (11.1)	0 (0.0)	9 (2.3)				
My gender influences my preference for snail consumption.	Student	86 (41.3)	93 (44.7)	21 (10.1)	7 (3.4)	1 (0.5)	208 (53.2)	114.242 (20)	<0.0001	<0.0001	0.270
	Trader	4 (14.8)	7 (25.9)	3 (11.1)	8 (29.6)	5 (18.5)	27 (6.9)				
	Civil servant	47 (43.1)	42 (38.5)	12 (11.0)	1 (0.9)	7 (6.4)	109 (27.9)				
	Artisan	1 (5.0)	4 (20.0)	4 (20.0)	8 (40.0)	3 (15.0)	20 (5.1)				
	Unemployed	9 (50.0)	7 (38.9)	1 (5.6)	1 (5.6)	0 (0.0)	18 (4.6)				
	Others	3 (33.3)	5 (55.6)	1 (11.1)	0 (0.0)	0 (0.0)	9 (2.3)				
Snails are more commonly consumed in low-income households in this area.	Student	52 (25.0)	90 (43.3)	34 (16.3)	24 (11.5)	8 (3.8)	208 (53.2)	69.875 (20)	<0.0001	<0.0001	0.211
	Trader	4 (14.8)	6 (22.2)	3 (11.1)	11 (40.7)	3 (11.1)	27 (6.9)				
	Civil servant	35 (32.1)	53 (48.6)	9 (8.3)	9 (8.3)	3 (2.8)	109 (27.9)				
	Artisan	1 (5.0)	3 (15.0)	3 (15.0)	8 (40.0)	5 (25.0)	20 (5.1)				
	Unemployed	5 (27.8)	5 (27.8)	3 (16.7)	5 (27.8)	0 (0.0)	18 (4.6)				
	Others	3 (33.3)	5 (55.6)	1 (11.1)	0 (0.0)	0 (0.0)	9 (2.3)				
I first learned about snail consumption from family traditions.	Student	35 (16.8)	53 (25.5)	34 (16.3)	68 (32.7)	18 (8.7)	208 (53.2)	36.249 (20)	0.0144	0.1438	0.152
	Trader	0 (0.0)	4 (14.8)	4 (14.8)	12 (44.4)	7 (25.9)	27 (6.9)				
	Civil servant	19 (17.4)	19 (17.4)	12 (11.0)	39 (35.8)	20 (18.3)	109 (27.9)				
	Artisan	1 (5.0)	1 (5.0)	4 (20.0)	8 (40.0)	6 (30.0)	20 (5.1)				
	Unemployed	1 (5.6)	7 (38.9)	3 (16.7)	6 (33.3)	1 (5.6)	18 (4.6)				
	Others	1 (11.1)	1 (11.1)	0 (0.0)	6 (66.7)	1 (11.1)	9 (2.3)				
The size of my household determines how much snail we consume weekly.	Student	34 (16.3)	60 (28.8)	34 (16.3)	65 (31.2)	15 (7.2)	208 (53.2)	37.681 (20)	0.0097	0.0968	0.155
	Trader	1 (3.7)	2 (7.4)	7 (25.9)	9 (33.3)	8 (29.6)	27 (6.9)				
	Civil servant	20 (18.3)	26 (23.9)	18 (16.5)	37 (33.9)	8 (7.3)	109 (27.9)				
	Artisan	2 (10.0)	3 (15.0)	2 (10.0)	8 (40.0)	5 (25.0)	20 (5.1)				
	Unemployed	4 (22.2)	2 (11.1)	5 (27.8)	7 (38.9)	0 (0.0)	18 (4.6)				
	Others	1 (11.1)	4 (44.4)	1 (11.1)	3 (33.3)	0 (0.0)	9 (2.3)				

Each response entry is reported as *n* (%); Values are expressed as frequency (n) and percentage (%); Values reported in the format n/N (%) in text indicate the number of respondents with the specified response (n), the total respondents in the relevant subgroup (N), and the corresponding percentage calculated as n divided by N multiplied by 100; Pearson *χ*^2^ with degrees of freedom, raw p-values, Bonferroni-adjusted *p*-values, and Cramér’s V are reported at item level. Bonferroni adjustment controls family-wise *α* = 0.05 across the 10 tests in each demographic-by-domain family.

In the occupation-based socioeconomic analyses (Table 3), associations surviving Bonferroni correction were observed for perceived nutritional benefits (adjusted *p* = 0.0092), employment/affordability (adjusted *p* = 0.0417), cultural or household preference (adjusted *p* = 0.0120), perceived cheaper protein (adjusted *p* < 0.001), gender-related preference (adjusted *p* < 0.001), and perceived dependence of low-income households (adjusted *p* < 0.001). For gender-related preference, 179/208 (86.1%) students and 89/109 (81.7%) civil servants disagreed or strongly disagreed, whereas 13/27 (48.1%) traders and 11/20 (55.0%) artisans agreed or strongly agreed. For the low-income-household item, 142/208 (68.3%) students and 88/109 (80.7%) civil servants disagreed or strongly disagreed, while 14/27 (51.9%) traders and 13/20 (65.0%) artisans agreed or strongly agreed.

Table 4 shows the Multiple Linear Regression Analysis of Socioeconomic Factors Related to Snail Consumption in Yenagoa Metropolis, Nigeria. While the Table 5: Summary of Model Fit Indices for the Four Questionnaire Domains in Yenagoa Metropolis, Nigeria. The regression model for the socioeconomic-consumption composite was statistically significant, *F* (5,385) = 12.525, *p* < 0.001, *R*^2^ = 0.140, adjusted *R*^2^ = 0.129 (Table 5). Marital status showed a positive independent association (*B* = 0.258, 95% CI 0.121 to 0.396, *β* = 0.222, *p* < 0.001), whereas education showed an inverse association (*B* = -0.272, 95% CI -0.368 to −0.176, *β* = −0.270, *p* < 0.001) (Table 4). Age, sex, and occupation were not statistically significant at *α* = 0.05. The model explained 14.0% of the variance in the composite outcome, indicating modest explanatory power. Accordingly, the result is presented as evidence that measured demographic characteristics account for only a limited portion of variation in the consumption-related domain.

**Table 4:** Multiple linear regression analysis of socioeconomic factors related to snail consumption in Yenagoa Metropolis, Nigeria.

Predictor	B	SE	β	t	p	95% CI for B	Tolerance	VIF
Constant	3.536	0.285	—	12.406	<0.001	2.976 to 4.096	—	—
Age	0.163	0.096	0.112	1.698	0.090	−0.026 to 0.351	0.513	1.949
Sex	−0.143	0.074	−0.098	−1.929	0.055	−0.289 to 0.003	0.873	1.146
Marital status	0.258	0.070	0.222	3.698	<0.001	0.121 to 0.396	0.621	1.610
Educational level	−0.272	0.049	−0.270	−5.585	<0.001	−0.368 to −0.176	0.953	1.050
Occupation	−0.053	0.032	−0.099	−1.652	0.099	−0.117 to 0.010	0.621	1.610

B, Unstandardized coefficient; SE, Standard error; *β*, Standardized coefficient; CI, 95% confidence interval; VIF, Variance inflation factor.

**Table 5:** Summary of model fit indices for the four questionnaire domains in Yenagoa Metropolis, Nigeria.

Model	Dependent variable	*R*	*R* ^2^	Adj. *R*^2^	F (df)	*p*	Durbin–Watson
1	Socioeconomic factors related to snail consumption	0.374	0.140	0.129	12.525 (5,385)	<0.001	1.577
2	Awareness, perceptions, and coping strategies concerning snail safety	0.347	0.120	0.107	8.768 (6,384)	<0.001	1.838
3	Perceived public-health implications and vulnerability	0.486	0.236	0.222	16.889 (7,383)	<0.001	1.952
4	Community-based interventions and policy measures	0.446	0.199	0.182	11.877 (8,382)	<0.001	1.697

*R*^2^ = coefficient of determination; adjusted *R*^2^ accounts for model complexity. Durbin–Watson values were close to 2 and did not suggest marked first-order residual autocorrelation.

#### 3.1.3 Awareness, perceptions, and coping strategies concerning snail safety

Six and seven display the complete education- and occupation-stratified distributions. Table 6 reports the multivariable regression, while Table 5 provides the corresponding model-fit summary. Awareness and protective behaviour were uneven. Concern about eating snails from areas near refuse dumps was comparatively high, whereas 39.9% reported that they did not check the source of snails before purchase. Familiarity with health risks associated with trace metals and confidence in selecting safe sources were mixed, and avoidance of unknown or roadside vendors was inconsistent. Trust in vendor hygiene was low: 53.2% reported no or only slight trust. By contrast, environmental cleanliness was considered very or extremely important by 63.0% of respondents. These distributions describe the achieved sample; education- and occupation-based association results are presented in Tables 7, 8.

**Table 6:** Multiple linear regression analysis of awareness, perceptions, and coping strategies concerning snail safety in Yenagoa Metropolis, Nigeria.

Predictor	B	SE	β	t	p	95% CI for B	Tolerance	VIF
Constant	1.760	0.402	—	4.378	<0.001	0.969 to 2.550	—	—
Age	0.058	0.115	0.034	0.504	0.614	−0.168 to 0.284	0.509	1.963
Sex	0.073	0.089	0.042	0.823	0.411	−0.102 to 0.248	0.864	1.157
Marital status	0.104	0.085	0.076	1.226	0.221	−0.063 to 0.271	0.600	1.667
Educational level	−0.017	0.060	−0.015	−0.289	0.773	−0.136 to 0.101	0.881	1.135
Occupation	−0.009	0.039	−0.014	−0.223	0.824	−0.085 to 0.067	0.617	1.621
Socioeconomic-consumption composite	0.370	0.061	0.314	6.084	<0.001	0.250 to 0.489	0.860	1.163

B, Unstandardized coefficient; SE, Standard error; *β*, Standardized coefficient; CI, 95% confidence interval; VIF, Variance inflation factor.

**Table 7:** Education-based association for awareness, perceptions, and coping strategies concerning snail safety in Yenagoa Metropolis, Nigeria.

Questionnaire item	Educational level	Not at all *n* (%)	Slightly *n* (%)	Moderately *n* (%)	Very *n* (%)	Extremely *n* (%)	Total *n* (%)	*χ*^2^ (df)	Raw *p*	Bonferroni- adjusted *p*	Cramér’s V
How aware are you of the potential for environmental pollution to affect snail safety?	No formal education	0 (0.0)	1 (50.0)	1 (50.0)	0 (0.0)	0 (0.0)	2 (0.5)	24.468 (16)	0.0798	0.7977	0.125
	Primary	1 (16.7)	0 (0.0)	1 (16.7)	2 (33.3)	2 (33.3)	6 (1.5)				
	Secondary	5 (12.5)	9 (22.5)	5 (12.5)	11 (27.5)	10 (25.0)	40 (10.2)				
	Tertiary	37 (17.7)	65 (31.1)	46 (22.0)	43 (20.6)	18 (8.6)	209 (53.5)				
	Postgraduate	19 (14.2)	25 (18.7)	30 (22.4)	38 (28.4)	22 (16.4)	134 (34.3)				
How concerned are you about eating snails from areas near refuse dumps?	No formal education	1 (50.0)	0 (0.0)	0 (0.0)	1 (50.0)	0 (0.0)	2 (0.5)	21.500 (16)	0.1601	1.0000	0.117
	Primary	1 (16.7)	0 (0.0)	3 (50.0)	0 (0.0)	2 (33.3)	6 (1.5)				
	Secondary	2 (5.0)	3 (7.5)	7 (17.5)	11 (27.5)	17 (42.5)	40 (10.2)				
	Tertiary	30 (14.4)	25 (12.0)	23 (11.0)	53 (25.4)	78 (37.3)	209 (53.5)				
	Postgraduate	12 (9.0)	12 (9.0)	23 (17.2)	43 (32.1)	44 (32.8)	134 (34.3)				
To what extent do you check the source of snails before buying?	No formal education	1 (50.0)	0 (0.0)	1 (50.0)	0 (0.0)	0 (0.0)	2 (0.5)	20.134 (16)	0.2142	1.0000	0.113
	Primary	1 (16.7)	1 (16.7)	2 (33.3)	1 (16.7)	1 (16.7)	6 (1.5)				
	Secondary	9 (22.5)	5 (12.5)	8 (20.0)	9 (22.5)	9 (22.5)	40 (10.2)				
	Tertiary	80 (38.3)	36 (17.2)	41 (19.6)	27 (12.9)	25 (12.0)	209 (53.5)				
	Postgraduate	65 (48.5)	16 (11.9)	27 (20.1)	17 (12.7)	9 (6.7)	134 (34.3)				
How much do you rely on personal observation to judge if snails are safe to eat?	No formal education	0 (0.0)	1 (50.0)	1 (50.0)	0 (0.0)	0 (0.0)	2 (0.5)	26.876 (16)	0.0429	0.4288	0.131
	Primary	0 (0.0)	3 (50.0)	2 (33.3)	1 (16.7)	0 (0.0)	6 (1.5)				
	Secondary	6 (15.0)	5 (12.5)	13 (32.5)	5 (12.5)	11 (27.5)	40 (10.2)				
	Tertiary	48 (23.0)	50 (23.9)	44 (21.1)	42 (20.1)	25 (12.0)	209 (53.5)				
	Postgraduate	38 (28.4)	21 (15.7)	41 (30.6)	20 (14.9)	14 (10.4)	134 (34.3)				
How familiar are you with the health risks associated with heavy metals in food?	No formal education	0 (0.0)	1 (50.0)	1 (50.0)	0 (0.0)	0 (0.0)	2 (0.5)	21.595 (16)	0.1568	1.0000	0.118
	Primary	1 (16.7)	1 (16.7)	2 (33.3)	2 (33.3)	0 (0.0)	6 (1.5)				
	Secondary	0 (0.0)	7 (17.5)	11 (27.5)	11 (27.5)	11 (27.5)	40 (10.2)				
	Tertiary	44 (21.1)	31 (14.8)	61 (29.2)	48 (23.0)	25 (12.0)	209 (53.5)				
	Postgraduate	21 (15.7)	15 (11.2)	37 (27.6)	38 (28.4)	23 (17.2)	134 (34.3)				
How often do you avoid buying snails from unknown or roadside vendors?	No formal education	1 (50.0)	0 (0.0)	0 (0.0)	1 (50.0)	0 (0.0)	2 (0.5)	25.549 (16)	0.0607	0.6071	0.128
	Primary	1 (16.7)	0 (0.0)	4 (66.7)	1 (16.7)	0 (0.0)	6 (1.5)				
	Secondary	5 (12.5)	5 (12.5)	7 (17.5)	12 (30.0)	11 (27.5)	40 (10.2)				
	Tertiary	57 (27.3)	40 (19.1)	38 (18.2)	38 (18.2)	36 (17.2)	209 (53.5)				
	Postgraduate	36 (26.9)	24 (17.9)	19 (14.2)	37 (27.6)	18 (13.4)	134 (34.3)				
How confident are you in your ability to choose clean and safe snail sources?	No formal education	0 (0.0)	0 (0.0)	1 (50.0)	0 (0.0)	1 (50.0)	2 (0.5)	30.212 (16)	0.0169	0.1693	0.139
	Primary	2 (20.0)	0 (0.0)	4 (80.0)	0 (0.0)	0 (0.0)	6 (1.5)				
	Secondary	3 (7.5)	3 (7.5)	11 (27.5)	10 (25.0)	13 (32.5)	40 (10.3)				
	Tertiary	35 (16.3)	50 (24.0)	58 (27.9)	40 (19.2)	26 (12.5)	208 (53.5)				
	Postgraduate	23 (17.2)	27 (20.1)	37 (27.6)	33 (24.6)	14 (10.4)	134 (34.4)				
How likely are you to discuss food safety concerns with friends or neighbors?	No formal education	0 (0.0)	0 (0.0)	2 (100.0)	0 (0.0)	0 (0.0)	2 (0.5)	27.538 (16)	0.0359	0.3588	0.133
	Primary	1 (16.7)	1 (16.7)	2 (33.3)	2 (33.3)	0 (0.0)	6 (1.5)				
	Secondary	1 (2.5)	4 (10.0)	9 (22.5)	11 (27.5)	15 (37.5)	40 (10.2)				
	Tertiary	25 (12.0)	38 (18.2)	52 (24.9)	68 (32.5)	26 (12.4)	209 (53.5)				
	Postgraduate	11 (8.2)	17 (12.7)	37 (27.6)	48 (35.8)	21 (15.7)	134 (34.3)				
How much trust do you place in the hygiene practices of snail vendors?	No formal education	0 (0.0)	2 (100.0)	0 (0.0)	0 (0.0)	0 (0.0)	2 (0.5)	33.761 (16)	0.0059	0.0585	0.147
	Primary	1 (16.7)	1 (16.7)	3 (50.0)	0 (0.0)	1 (16.7)	6 (1.5)				
	Secondary	2 (5.0)	10 (25.0)	8 (20.0)	11 (27.5)	9 (22.5)	40 (10.2)				
	Tertiary	48 (23.0)	67 (32.1)	58 (27.8)	22 (10.5)	14 (6.7)	209 (53.5)				
	Postgraduate	36 (26.9)	41 (30.6)	31 (23.1)	16 (11.9)	10 (7.5)	134 (34.3)				
How important is environmental cleanliness to your decision to consume snails?	No formal education	0 (0.0)	0 (0.0)	0 (0.0)	1 (50.0)	1 (50.0)	2 (0.5)	22.071 (16)	0.1409	1.0000	0.119
	Primary	1 (16.7)	1 (16.7)	3 (50.0)	1 (16.7)	0 (0.0)	6 (1.5)				
	Secondary	1 (2.5)	8 (20.0)	10 (25.0)	8 (20.0)	13 (32.5)	40 (10.2)				
	Tertiary	21 (10.0)	19 (9.1)	33 (15.8)	75 (35.9)	61 (29.2)	209 (53.5)				
	Postgraduate	11 (8.2)	11 (8.2)	26 (19.4)	56 (41.8)	30 (22.4)	134 (34.3)				

Footnote: Each response entry is reported as *n* (%); Values reported in the format n/N (%) in text indicate the number of respondents with the specified response (n), the total respondents in the relevant subgroup (N), and the corresponding percentage calculated as n divided by N multiplied by 100; Pearson *χ*^2^ with degrees of freedom, raw p-values, Bonferroni-adjusted *p*-values, and Cramér’s V are reported at item level. Bonferroni adjustment controls family-wise *α* = 0.05 across the 10 tests in each demographic-by-domain family.

**Table 8:** Occupation-based association for awareness, perceptions, and coping strategies concerning snail safety in Yenagoa Metropolis, Nigeria.

Questionnaire item	Occupation	Not at all *n* (%)	Slightly*n* (%)	Moderately *n* (%)	Very *n* (%)	Extremely *n* (%)	Total *n* (%)	*χ*^2^ (df)	Raw *p*	Bonferroni- adjusted *p*	Cramér’s V
How aware are you of the potential for environmental pollution to affect snail safety?	Student	42 (20.2)	61 (29.3)	41 (19.7)	47 (22.6)	17 (8.2)	208 (53.2)	48.462 (20)	0.0004	0.0037	0.176
	Trader	2 (7.4)	3 (11.1)	6 (22.2)	11 (40.7)	5 (18.5)	27 (6.9)				
	Civil servant	13 (11.9)	26 (23.9)	26 (23.9)	24 (22.0)	20 (18.3)	109 (27.9)				
	Artisan	1 (5.0)	0 (0.0)	4 (20.0)	8 (40.0)	7 (35.0)	20 (5.1)				
	Unemployed	1 (5.6)	8 (44.4)	5 (27.8)	4 (22.2)	0 (0.0)	18 (4.6)				
	Others	3 (33.3)	2 (22.2)	1 (11.1)	0 (0.0)	3 (33.3)	9 (2.3)				
How concerned are you about eating snails from areas near refuse dumps?	Student	31 (14.9)	21 (10.1)	33 (15.9)	52 (25.0)	71 (34.1)	208 (53.2)	23.592 (20)	0.2607	1.0000	0.123
	Trader	0 (0.0)	4 (14.8)	6 (22.2)	7 (25.9)	10 (37.0)	27 (6.9)				
	Civil servant	9 (8.3)	12 (11.0)	12 (11.0)	35 (32.1)	41 (37.6)	109 (27.9)				
	Artisan	1 (5.0)	0 (0.0)	3 (15.0)	7 (35.0)	9 (45.0)	20 (5.1)				
	Unemployed	5 (27.8)	3 (16.7)	1 (5.6)	4 (22.2)	5 (27.8)	18 (4.6)				
	Others	0 (0.0)	0 (0.0)	1 (11.1)	3 (33.3)	5 (55.6)	9 (2.3)				
To what extent do you check the source of snails before buying?	Student	85 (40.9)	31 (14.9)	43 (20.7)	29 (13.9)	20 (9.6)	208 (53.2)	49.835 (20)	0.0002	0.0023	0.179
	Trader	6 (22.2)	5 (18.5)	4 (14.8)	5 (18.5)	7 (25.9)	27 (6.9)				
	Civil servant	51 (46.8)	10 (9.2)	24 (22.0)	11 (10.1)	13 (11.9)	109 (27.9)				
	Artisan	1 (5.0)	3 (15.0)	5 (25.0)	8 (40.0)	3 (15.0)	20 (5.1)				
	Unemployed	8 (44.4)	8 (44.4)	0 (0.0)	1 (5.6)	1 (5.6)	18 (4.6)				
	Others	5 (55.6)	1 (11.1)	3 (33.3)	0 (0.0)	0 (0.0)	9 (2.3)				
How much do you rely on personal observation to judge if snails are safe to eat?	Student	49 (23.6)	45 (21.6)	55 (26.4)	38 (18.3)	21 (10.1)	208 (53.2)	25.381 (20)	0.1872	1.0000	0.127
	Trader	4 (14.8)	5 (18.5)	6 (22.2)	5 (18.5)	7 (25.9)	27 (6.9)				
	Civil servant	32 (29.4)	20 (18.3)	22 (20.2)	20 (18.3)	15 (13.8)	109 (27.9)				
	Artisan	0 (0.0)	3 (15.0)	9 (45.0)	4 (20.0)	4 (20.0)	20 (5.1)				
	Unemployed	4 (22.2)	6 (33.3)	6 (33.3)	1 (5.6)	1 (5.6)	18 (4.6)				
	Others	3 (33.3)	1 (11.1)	3 (33.3)	0 (0.0)	2 (22.2)	9 (2.3)				
How familiar are you with the health risks associated with heavy metals in food?	Student	33 (15.9)	37 (17.8)	61 (29.3)	46 (22.1)	31 (14.9)	208 (53.2)	25.184 (20)	0.1945	1.0000	0.127
	Trader	3 (11.1)	3 (11.1)	9 (33.3)	7 (25.9)	5 (18.5)	27 (6.9)				
	Civil servant	25 (22.9)	7 (6.4)	28 (25.7)	32 (29.4)	17 (15.6)	109 (27.9)				
	Artisan	1 (5.0)	1 (5.0)	6 (30.0)	8 (40.0)	4 (20.0)	20 (5.1)				
	Unemployed	3 (16.7)	6 (33.3)	5 (27.8)	4 (22.2)	0 (0.0)	18 (4.6)				
	Others	1 (11.1)	1 (11.1)	3 (33.3)	2 (22.2)	2 (22.2)	9 (2.3)				
How often do you avoid buying snails from unknown or roadside vendors?	Student	52 (25.0)	43 (20.7)	32 (15.4)	46 (22.1)	35 (16.8)	208 (53.2)	32.262 (20)	0.0406	0.4059	0.144
	Trader	4 (14.8)	2 (7.4)	10 (37.0)	7 (25.9)	4 (14.8)	27 (6.9)				
	Civil servant	33 (30.3)	20 (18.3)	17 (15.6)	20 (18.3)	19 (17.4)	109 (27.9)				
	Artisan	1 (5.0)	1 (5.0)	3 (15.0)	10 (50.0)	5 (25.0)	20 (5.1)				
	Unemployed	7 (38.9)	3 (16.7)	3 (16.7)	3 (16.7)	2 (11.1)	18 (4.6)				
	Others	3 (33.3)	0 (0.0)	3 (33.3)	3 (33.3)	0 (0.0)	9 (2.3)				
How confident are you in your ability to choose clean and safe snail sources?	Student	32 (15.0)	46 (22.2)	62 (30.0)	42 (20.3)	26 (12.6)	208 (53.2)	29.137 (20)	0.0851	0.8510	0.137
	Trader	2 (7.4)	3 (11.1)	10 (37.0)	5 (18.5)	7 (25.9)	27 (6.9)				
	Civil servant	21 (19.3)	21 (19.3)	29 (26.6)	26 (23.9)	12 (11.0)	109 (28.0)				
	Artisan	2 (5.3)	1 (5.3)	4 (21.1)	7 (36.8)	6 (31.6)	20 (4.9)				
	Unemployed	4 (22.2)	8 (44.4)	4 (22.2)	1 (5.6)	1 (5.6)	18 (4.6)				
	Others	2 (22.2)	1 (11.1)	2 (22.2)	2 (22.2)	2 (22.2)	9 (2.3)				
How likely are you to discuss food safety concerns with friends or neighbors?	Student	24 (11.5)	38 (18.3)	54 (26.0)	65 (31.2)	27 (13.0)	208 (53.2)	27.176 (20)	0.1304	1.0000	0.132
	Trader	3 (11.1)	3 (11.1)	9 (33.3)	6 (22.2)	6 (22.2)	27 (6.9)				
	Civil servant	6 (5.5)	18 (16.5)	22 (20.2)	43 (39.4)	20 (18.3)	109 (27.9)				
	Artisan	1 (5.0)	0 (0.0)	7 (35.0)	5 (25.0)	7 (35.0)	20 (5.1)				
	Unemployed	3 (16.7)	1 (5.6)	6 (33.3)	6 (33.3)	2 (11.1)	18 (4.6)				
	Others	1 (11.1)	0 (0.0)	4 (44.4)	4 (44.4)	0 (0.0)	9 (2.3)				
How much trust do you place in the hygiene practices of snail vendors?	Student	47 (22.6)	71 (34.1)	49 (23.6)	25 (12.0)	16 (7.7)	208 (53.2)	56.166 (20)	<0.0001	0.0003	0.190
	Trader	1 (3.7)	6 (22.2)	10 (37.0)	7 (25.9)	3 (11.1)	27 (6.9)				
	Civil servant	28 (25.7)	38 (34.9)	27 (24.8)	9 (8.3)	7 (6.4)	109 (27.9)				
	Artisan	1 (5.0)	1 (5.0)	5 (25.0)	5 (25.0)	8 (40.0)	20 (5.1)				
	Unemployed	7 (38.9)	3 (16.7)	5 (27.8)	3 (16.7)	0 (0.0)	18 (4.6)				
	Others	3 (33.3)	2 (22.2)	4 (44.4)	0 (0.0)	0 (0.0)	9 (2.3)				
How important is environmental cleanliness to your decision to consume snails?	Student	20 (9.6)	27 (13.0)	29 (13.9)	75 (36.1)	57 (27.4)	208 (53.2)	28.426 (20)	0.0997	0.9969	0.135
	Trader	1 (3.7)	4 (14.8)	8 (29.6)	7 (25.9)	7 (25.9)	27 (6.9)				
	Civil servant	10 (9.2)	8 (7.3)	17 (15.6)	42 (38.5)	32 (29.4)	109 (27.9)				
	Artisan	1 (5.0)	0 (0.0)	8 (40.0)	6 (30.0)	5 (25.0)	20 (5.1)				
	Unemployed	2 (11.1)	0 (0.0)	7 (38.9)	7 (38.9)	2 (11.1)	18 (4.6)				
	Others	0 (0.0)	0 (0.0)	3 (33.3)	4 (44.4)	2 (22.2)	9 (2.3)				

Each response entry is reported as *n* (%); Values reported in the format n/N (%) in text indicate the number of respondents with the specified response (n), the total respondents in the relevant subgroup (N), and the corresponding percentage calculated as n divided by N multiplied by 100; Pearson *χ*^2^ with degrees of freedom, raw *p*-values, Bonferroni-adjusted p-values, and Cramér’s V are reported at item level. Bonferroni adjustment controls family-wise *α* = 0.05 across the 10 tests in each demographic-by-domain family.

After Bonferroni correction across the 10 occupation-based awareness tests, significant associations remained for awareness that environmental pollution can affect snail safety (adjusted *p* = 0.0037), checking the source before purchase (adjusted *p* = 0.0023), and trust in vendor hygiene (adjusted *p* = 0.0003). As shown in Table 8, Very or Extremely high pollution awareness was reported by 15/20 (75.0%) artisans and 16/27 (59.3%) traders, compared with 64/208 (30.8%) students. For source checking, 116/208 (55.8%) students, 61/109 (56.0%) civil servants, and 16/18 (88.9%) unemployed respondents selected Not at all or Slightly, whereas 11/20 (55.0%) artisans selected Very or Extremely. For trust in vendor hygiene, 118/208 (56.7%) students and 66/109 (60.6%) civil servants reported Not at all or Slightly, compared with 13/20 (65.0%) artisans who reported Very or Extremely. The raw association for avoiding roadside vendors did not survive Bonferroni correction. None of the 10 education-based awareness associations in Table 7 remained statistically significant after Bonferroni correction. The smallest adjusted *p*-value was for trust in vendor hygiene (raw *p* = 0.0059; adjusted *p* = 0.0585).

Table 6: Multiple Linear Regression Analysis of Awareness, Perceptions, and Coping Strategies Concerning Snail Safety in Yenagoa Metropolis, Nigeria. The awareness regression model was significant overall, *F*(6,384) = 8.768, *p* < 0.001, but explained 12.0% of variance (adjusted *R*^2^ = 0.107) (Table 5). The socioeconomic-consumption composite was the only statistically significant independent correlate (B = 0.370, 95% CI 0.250 to 0.489, *β* = 0.314, *p* < 0.001; Table 6). Age, sex, marital status, education, and occupation were not statistically significant. The modest *R*^2^ indicates that most variation in awareness was not accounted for by the included predictors.

#### 3.1.4 Perceived public-health implications and vulnerability

Tables 9, 10 show the complete education- and occupation-stratified distributions across the five truth-rating categories on perceived public-health implications and vulnerability related to potentially contaminated snail consumption. Table 11 presents the multivariable regression and Table 5 summarises model fit; these items measure perceived risk and vulnerability. Perceptions of vulnerability were mixed across the first four items, including perceived exposure of low-income families, susceptibility of children, unsafe-source purchasing among informal workers, and household ability to verify snail safety. Responses shifted more toward agreement for statements concerning inadequate health education, limited awareness among vulnerable groups, restricted access to safety information, and the perspective that trace-element exposure from contaminated snails could constitute a public-health threat. In the education-based public-health-perception analyses (Table 9), perceived exposure of low-income families and perceived vulnerability of children also remained significant after Bonferroni correction (both adjusted *p* < 0.001). For each statement, 24/40 (60.0%) respondents with secondary education selected Mostly true or Completely true. The corresponding high-endorsement frequencies were 64/209 (30.6%) among tertiary and 43/134 (32.1%) among postgraduate respondents for low-income-family exposure, and 54/209 (25.8%) and 30/134 (22.4%), respectively, for child vulnerability. The cost-of-safer-food item was not significant after correction.

**Table 9:** Education-based association for perceived public-health implications and vulnerability in Yenagoa Metropolis, Nigeria.

Questionnaire item	Educational level	Not true *n* (%)	Slightly true *n* (%)	Moderately true *n* (%)	Mostly true *n* (%)	Completely true n (%)	Total *n* (%)	*χ*^2^ (df)	Raw *p*	Bonferroni- adjusted *p*	Cramér’s V
Low-income families in this area are more exposed to contaminated snail meat	No formal education	0 (0.0)	0 (0.0)	2 (100.0)	0 (0.0)	0 (0.0)	2 (0.5)	57.019 (16)	<0.0001	<0.0001	0.191
	Primary	0 (0.0)	2 (33.3)	0 (0.0)	3 (50.0)	1 (16.7)	6 (1.5)				
	Secondary	6 (15.0)	3 (7.5)	7 (17.5)	11 (27.5)	13 (32.5)	40 (10.2)				
	Tertiary	51 (24.4)	45 (21.5)	49 (23.4)	53 (25.4)	11 (5.3)	209 (53.5)				
	Postgraduate	51 (38.1)	24 (17.9)	16 (11.9)	27 (20.1)	16 (11.9)	134 (34.3)				
Children who consume snails may be more vulnerable to trace metal toxicity.	No formal education	0 (0.0)	0 (0.0)	1 (50.0)	1 (50.0)	0 (0.0)	2 (0.5)	61.337 (16)	<0.0001	<0.0001	0.198
	Primary	1 (16.7)	1 (16.7)	2 (33.3)	0 (0.0)	2 (33.3)	6 (1.5)				
	Secondary	8 (20.0)	1 (2.5)	7 (17.5)	10 (25.0)	14 (35.0)	40 (10.2)				
	Tertiary	44 (21.1)	54 (25.8)	57 (27.3)	45 (21.5)	9 (4.3)	209 (53.5)				
	Postgraduate	36 (26.9)	41 (30.6)	27 (20.1)	21 (15.7)	9 (6.7)	134 (34.3)				
Informal workers are more likely to consume snails from unsafe sources due to affordability.	No formal education	0 (0.0)	0 (0.0)	1 (50.0)	1 (50.0)	0 (0.0)	2 (0.5)	21.402 (16)	0.1636	1.0000	0.117
	Primary	0 (0.0)	3 (50.0)	1 (16.7)	2 (33.3)	0 (0.0)	6 (1.5)				
	Secondary	5 (12.5)	5 (12.5)	10 (25.0)	13 (32.5)	7 (17.5)	40 (10.2)				
	Tertiary	41 (19.6)	57 (27.3)	55 (26.3)	42 (20.1)	14 (6.7)	209 (53.5)				
	Postgraduate	34 (25.4)	37 (27.6)	25 (18.7)	28 (20.9)	10 (7.5)	134 (34.3)				
My household cannot always verify the safety of snails before consumption.	No formal education	0 (0.0)	1 (50.0)	1 (50.0)	0 (0.0)	0 (0.0)	2 (0.5)	14.557 (16)	0.5573	1.0000	0.096
	Primary	0 (0.0)	2 (33.3)	2 (33.3)	1 (16.7)	1 (16.7)	6 (1.5)				
	Secondary	6 (15.0)	2 (5.0)	13 (32.5)	11 (27.5)	8 (20.0)	40 (10.2)				
	Tertiary	43 (20.6)	45 (21.5)	49 (23.4)	47 (22.5)	25 (12.0)	209 (53.5)				
	Postgraduate	21 (15.7)	26 (19.4)	34 (25.4)	30 (22.4)	23 (17.2)	134 (34.3)				
Poor access to health education increases snail-related health risks.	No formal education	0 (0.0)	0 (0.0)	0 (0.0)	2 (100.0)	0 (0.0)	2 (0.5)	17.090 (16)	0.3798	1.0000	0.105
	Primary	0 (0.0)	1 (16.7)	0 (0.0)	4 (66.7)	1 (16.7)	6 (1.5)				
	Secondary	4 (10.0)	2 (5.0)	8 (20.0)	14 (35.0)	12 (30.0)	40 (10.2)				
	Tertiary	19 (9.1)	37 (17.7)	51 (24.4)	57 (27.3)	45 (21.5)	209 (53.5)				
	Postgraduate	16 (11.9)	16 (11.9)	30 (22.4)	43 (32.1)	29 (21.6)	134 (34.3)				
Vulnerable groups are less aware of the risks of contaminated snails.	No formal education	1 (50.0)	0 (0.0)	0 (0.0)	1 (50.0)	0 (0.0)	2 (0.5)	21.454 (16)	0.1617	1.0000	0.117
	Primary	0 (0.0)	1 (16.7)	4 (66.7)	0 (0.0)	1 (16.7)	6 (1.5)				
	Secondary	2 (5.0)	4 (10.0)	14 (35.0)	13 (32.5)	7 (17.5)	40 (10.2)				
	Tertiary	14 (6.7)	32 (15.3)	51 (24.4)	60 (28.7)	52 (24.9)	209 (53.5)				
	Postgraduate	13 (9.7)	21 (15.7)	24 (17.9)	37 (27.6)	39 (29.1)	134 (34.3)				
The cost of safer food options compels many to consume unverified snail sources.	No formal education	0 (0.0)	0 (0.0)	0 (0.0)	1 (50.0)	1 (50.0)	2 (0.5)	27.998 (16)	0.0316	0.3164	0.134
	Primary	0 (0.0)	1 (16.7)	3 (50.0)	1 (16.7)	1 (16.7)	6 (1.5)				
	Secondary	5 (12.5)	2 (5.0)	6 (15.0)	19 (47.5)	8 (20.0)	40 (10.2)				
	Tertiary	39 (18.7)	35 (16.7)	59 (28.2)	52 (24.9)	24 (11.5)	209 (53.5)				
	Postgraduate	28 (20.9)	21 (15.7)	25 (18.7)	31 (23.1)	29 (21.6)	134 (34.3)				
Nutritional dependence on snails puts economically disadvantaged groups at greater risk.	No formal education	0 (0.0)	1 (50.0)	0 (0.0)	1 (50.0)	0 (0.0)	2 (0.5)	23.174 (16)	0.1092	1.0000	0.122
	Primary	0 (0.0)	2 (33.3)	2 (33.3)	1 (16.7)	1 (16.7)	6 (1.5)				
	Secondary	5 (12.5)	8 (20.0)	6 (15.0)	11 (27.5)	10 (25.0)	40 (10.2)				
	Tertiary	41 (19.6)	35 (16.7)	72 (34.4)	39 (18.7)	22 (10.5)	209 (53.5)				
	Postgraduate	37 (27.6)	22 (16.4)	36 (26.9)	26 (19.4)	13 (9.7)	134 (34.3)				
There is limited information available to vulnerable groups about snail safety.	No formal education	0 (0.0)	0 (0.0)	0 (0.0)	1 (50.0)	1 (50.0)	2 (0.5)	15.053 (16)	0.5208	1.0000	0.098
	Primary	0 (0.0)	1 (16.7)	1 (16.7)	2 (33.3)	2 (33.3)	6 (1.5)				
	Secondary	1 (2.5)	4 (10.0)	8 (20.0)	9 (22.5)	18 (45.0)	40 (10.2)				
	Tertiary	13 (6.2)	31 (14.8)	48 (23.0)	70 (33.5)	47 (22.5)	209 (53.5)				
	Postgraduate	8 (6.0)	14 (10.4)	21 (15.7)	48 (35.8)	43 (32.1)	134 (34.3)				
Trace metal exposure from snails is a silent public health threat in this community.	No formal education	0 (0.0)	0 (0.0)	0 (0.0)	1 (50.0)	1 (50.0)	2 (0.5)	12.624 (16)	0.7000	1.0000	0.090
	Primary	0 (0.0)	2 (33.3)	1 (16.7)	2 (33.3)	1 (16.7)	6 (1.5)				
	Secondary	4 (10.0)	5 (12.5)	8 (20.0)	13 (32.5)	10 (25.0)	40 (10.2)				
	Tertiary	18 (8.6)	35 (16.7)	45 (21.5)	55 (26.3)	56 (26.8)	209 (53.5)				
	Postgraduate	8 (6.0)	15 (11.2)	29 (21.6)	30 (22.4)	52 (38.8)	134 (34.3)				

Each response entry is reported as *n* (%); Values are expressed as frequency (n) and percentage (%); Values reported in the format n/N (%) in text indicate the number of respondents with the specified response (n), the total respondents in the relevant subgroup (N), and the corresponding percentage calculated as n divided by N multiplied by 100; Pearson *χ*^2^ with degrees of freedom, raw *p*-values, Bonferroni-adjusted p-values, and Cramér’s V are reported at item level. Bonferroni adjustment controls family-wise *α* = 0.05 across the 10 tests in each demographic-by-domain family.

**Table 10:** Occupation-based association for perceived public-health implications and vulnerability in Yenagoa Metropolis, Nigeria.

Questionnaire item	Occupation	Not true *n* (%)	Slightly true *n* (%)	Moderately true *n* (%)	Mostly true *n* (%)	Completely true *n* (%)	Total *n* (%)	*χ*^2^ (df)	Raw *p*	Bonferroni- adjusted *p*	Cramér’s V
Low-income families in this area are more exposed to contaminated snail meat.	Student	48 (23.1)	45 (21.6)	49 (23.6)	52 (25.0)	14 (6.7)	208 (53.2)	67.084 (20)	<0.0001	<0.0001	0.207
	Trader	6 (22.2)	4 (14.8)	2 (7.4)	10 (37.0)	5 (18.5)	27 (6.9)				
	Civil servant	43 (39.4)	22 (20.2)	9 (8.3)	23 (21.1)	12 (11.0)	109 (27.9)				
	Artisan	0 (0.0)	1 (5.0)	4 (20.0)	7 (35.0)	8 (40.0)	20 (5.1)				
	Unemployed	9 (50.0)	0 (0.0)	6 (33.3)	2 (11.1)	1 (5.6)	18 (4.6)				
	Others	2 (22.2)	2 (22.2)	4 (44.4)	0 (0.0)	1 (11.1)	9 (2.3)				
Children who consume snails may be more vulnerable to trace metal toxicity.	Student	44 (21.2)	52 (25.0)	60 (28.8)	40 (19.2)	12 (5.8)	208 (53.2)	63.580 (20)	<0.0001	<0.0001	0.202
	Trader	6 (22.2)	4 (14.8)	1 (3.7)	8 (29.6)	8 (29.6)	27 (6.9)				
	Civil servant	25 (22.9)	36 (33.0)	23 (21.1)	19 (17.4)	6 (5.5)	109 (27.9)				
	Artisan	0 (0.0)	2 (10.0)	6 (30.0)	6 (30.0)	6 (30.0)	20 (5.1)				
	Unemployed	9 (50.0)	2 (11.1)	2 (11.1)	4 (22.2)	1 (5.6)	18 (4.6)				
	Others	5 (55.6)	1 (11.1)	2 (22.2)	0 (0.0)	1 (11.1)	9 (2.3)				
Informal workers are more likely to consume snails from unsafe sources due to affordability.	Student	41 (19.7)	57 (27.4)	49 (23.6)	41 (19.7)	20 (9.6)	208 (53.2)	36.292 (20)	0.0142	0.1421	0.152
	Trader	4 (14.8)	7 (25.9)	3 (11.1)	11 (40.7)	2 (7.4)	27 (6.9)				
	Civil servant	26 (23.9)	33 (30.3)	22 (20.2)	23 (21.1)	5 (4.6)	109 (27.9)				
	Artisan	0 (0.0)	1 (5.0)	8 (40.0)	7 (35.0)	4 (20.0)	20 (5.1)				
	Unemployed	7 (38.9)	2 (11.1)	7 (38.9)	2 (11.1)	0 (0.0)	18 (4.6)				
	Others	2 (22.2)	2 (22.2)	3 (33.3)	2 (22.2)	0 (0.0)	9 (2.3)				
My household cannot always verify the safety of snails before consumption.	Student	50 (24.0)	43 (20.7)	50 (24.0)	42 (20.2)	23 (11.1)	208 (53.2)	36.687 (20)	0.0128	0.1275	0.153
	Trader	5 (18.5)	4 (14.8)	5 (18.5)	6 (22.2)	7 (25.9)	27 (6.9)				
	Civil servant	11 (10.1)	24 (22.0)	26 (23.9)	25 (22.9)	23 (21.1)	109 (27.9)				
	Artisan	0 (0.0)	1 (5.0)	10 (50.0)	7 (35.0)	2 (10.0)	20 (5.1)				
	Unemployed	3 (16.7)	4 (22.2)	4 (22.2)	5 (27.8)	2 (11.1)	18 (4.6)				
	Others	1 (11.1)	0 (0.0)	4 (44.4)	4 (44.4)	0 (0.0)	9 (2.3)				
Poor access to health education increases snail-related health risks.	Student	22 (10.6)	32 (15.4)	54 (26.0)	52 (25.0)	48 (23.1)	208 (53.2)	27.788 (20)	0.1145	1.0000	0.133
	Trader	3 (11.1)	1 (3.7)	7 (25.9)	8 (29.6)	8 (29.6)	27 (6.9)				
	Civil servant	9 (8.3)	18 (16.5)	21 (19.3)	39 (35.8)	22 (20.2)	109 (27.9)				
	Artisan	0 (0.0)	1 (5.0)	3 (15.0)	11 (55.0)	5 (25.0)	20 (5.1)				
	Unemployed	4 (22.2)	1 (5.6)	2 (11.1)	9 (50.0)	2 (11.1)	18 (4.6)				
	Others	1 (11.1)	3 (33.3)	2 (22.2)	1 (11.1)	2 (22.2)	9 (2.3)				
Vulnerable groups are less aware of the risks of contaminated snails.	Student	14 (6.7)	39 (18.8)	47 (22.6)	59 (28.4)	49 (23.6)	208 (53.2)	21.177 (20)	0.3868	1.0000	0.116
	Trader	4 (14.8)	3 (11.1)	8 (29.6)	3 (11.1)	9 (33.3)	27 (6.9)				
	Civil servant	8 (7.3)	10 (9.2)	26 (23.9)	34 (31.2)	31 (28.4)	109 (27.9)				
	Artisan	0 (0.0)	1 (5.0)	7 (35.0)	7 (35.0)	5 (25.0)	20 (5.1)				
	Unemployed	3 (16.7)	4 (22.2)	4 (22.2)	5 (27.8)	2 (11.1)	18 (4.6)				
	Others	1 (11.1)	1 (11.1)	1 (11.1)	3 (33.3)	3 (33.3)	9 (2.3)				
The cost of safer food options compels many to consume unverified snail sources.	Student	39 (18.8)	39 (18.8)	53 (25.5)	47 (22.6)	30 (14.4)	208 (53.2)	31.104 (20)	0.0538	0.5383	0.141
	Trader	4 (14.8)	1 (3.7)	8 (29.6)	8 (29.6)	6 (22.2)	27 (6.9)				
	Civil servant	22 (20.2)	13 (11.9)	25 (22.9)	31 (28.4)	18 (16.5)	109 (27.9)				
	Artisan	0 (0.0)	1 (5.0)	2 (10.0)	11 (55.0)	6 (30.0)	20 (5.1)				
	Unemployed	6 (33.3)	2 (11.1)	3 (16.7)	6 (33.3)	1 (5.6)	18 (4.6)				
	Others	1 (11.1)	3 (33.3)	2 (22.2)	1 (11.1)	2 (22.2)	9 (2.3)				
Nutritional dependence on snails puts economically disadvantaged groups at greater risk.	Student	43 (20.7)	36 (17.3)	65 (31.2)	41 (19.7)	23 (11.1)	208 (53.2)	39.035 (20)	0.0066	0.0660	0.158
	Trader	4 (14.8)	4 (14.8)	8 (29.6)	3 (11.1)	8 (29.6)	27 (6.9)				
	Civil servant	28 (25.7)	15 (13.8)	35 (32.1)	22 (20.2)	9 (8.3)	109 (27.9)				
	Artisan	0 (0.0)	4 (20.0)	2 (10.0)	8 (40.0)	6 (30.0)	20 (5.1)				
	Unemployed	5 (27.8)	6 (33.3)	5 (27.8)	2 (11.1)	0 (0.0)	18 (4.6)				
	Others	3 (33.3)	3 (33.3)	1 (11.1)	2 (22.2)	0 (0.0)	9 (2.3)				
There is limited information available to vulnerable groups about snail safety.	Student	13 (6.2)	33 (15.9)	48 (23.1)	65 (31.2)	49 (23.6)	208 (53.2)	34.986 (20)	0.0202	0.2018	0.150
	Trader	3 (11.1)	2 (7.4)	2 (7.4)	8 (29.6)	12 (44.4)	27 (6.9)				
	Civil servant	2 (1.8)	9 (8.3)	16 (14.7)	45 (41.3)	37 (33.9)	109 (27.9)				
	Artisan	0 (0.0)	1 (5.0)	6 (30.0)	5 (25.0)	8 (40.0)	20 (5.1)				
	Unemployed	3 (16.7)	2 (11.1)	5 (27.8)	5 (27.8)	3 (16.7)	18 (4.6)				
	Others	1 (11.1)	3 (33.3)	1 (11.1)	2 (22.2)	2 (22.2)	9 (2.3)				
Trace metal exposure from snails is a silent public health threat in this community.	Student	15 (7.2)	38 (18.3)	43 (20.7)	51 (24.5)	61 (29.3)	208 (53.2)	27.826 (20)	0.1136	1.0000	0.133
	Trader	5 (18.5)	1 (3.7)	3 (11.1)	8 (29.6)	10 (37.0)	27 (6.9)				
	Civil servant	6 (5.5)	11 (10.1)	25 (22.9)	29 (26.6)	38 (34.9)	109 (27.9)				
	Artisan	0 (0.0)	2 (10.0)	4 (20.0)	7 (35.0)	7 (35.0)	20 (5.1)				
	Unemployed	4 (22.2)	2 (11.1)	6 (33.3)	4 (22.2)	2 (11.1)	18 (4.6)				
	Others	0 (0.0)	3 (33.3)	2 (22.2)	2 (22.2)	2 (22.2)	9 (2.3)				

Each response entry is reported as n (%); Values are expressed as frequency (n) and percentage (%); Values reported in the format n/N (%) in text indicate the number of respondents with the specified response (n), the total respondents in the relevant subgroup (N), and the corresponding percentage calculated as n divided by N multiplied by 100; Pearson *χ*^2^ with degrees of freedom, raw p-values, Bonferroni-adjusted p-values, and Cramér’s V are reported at item level. Bonferroni adjustment controls family-wise *α* = 0.05 across the 10 tests in each demographic-by-domain family.

**Table 11:** Multiple linear regression analysis of perceived public-health implications and vulnerability in Yenagoa Metropolis, Nigeria.

Predictor	B	SE	β	t	p	95% CI for B	Tolerance	VIF
Constant	1.050	0.389	—	2.698	0.007	0.285 to 1.815	—	—
Age	0.159	0.106	0.094	1.502	0.134	−0.049 to 0.367	0.509	1.965
Sex	−0.126	0.082	−0.074	−1.534	0.126	−0.288 to 0.036	0.863	1.159
Marital status	0.055	0.078	0.041	0.703	0.482	−0.099 to 0.209	0.597	1.674
Educational level	−0.068	0.056	−0.058	−1.223	0.222	−0.178 to 0.041	0.881	1.135
Occupation	−0.055	0.036	−0.087	−1.537	0.125	−0.125 to 0.015	0.616	1.622
Socioeconomic-consumption composite	0.260	0.059	0.224	4.429	<0.001	0.145 to 0.376	0.784	1.275
Awareness composite	0.301	0.047	0.304	6.407	<0.001	0.208 to 0.393	0.879	1.137

B, Unstandardized coefficient; SE, Standard error; *β*, Standardized coefficient; CI, 95% confidence interval; VIF, Variance inflation factor.

After Bonferroni adjustment of the ten occupation-based public-health tests, only perceived exposure of low-income families and perceived vulnerability of children remained statistically significant (both adjusted *p* < 0.001). In Table 10, 12/20 (75.0%) artisans rated the low-income-family statement as Mostly true or completely true, compared with 35/109 (32.1%) civil servants; 65/109 (59.6%) civil servants instead selected Not true or Slightly true. For perceived child vulnerability, 12/20 (60.0%) artisans and 16/27 (59.3%) traders selected Mostly true or completely true, whereas 61/109 (56.0%) civil servants selected Not true or Slightly true. Other occupation-based raw *p*-values did not survive family-wise correction.

**Table 12:** Reliability analysis of questionnaire constructs.

Construct	No. of Items	Cronbach’s Alpha	Interpretation
Socioeconomic factors related to snail consumption	10	0.806	Good
Awareness, perceptions, and coping strategies concerning snail safety	10	0.849	Good
Perceived public-health implications and vulnerability	10	0.855	Good
Community-based interventions and policy measures	10	0.948	Excellent
Combined questionnaire	40	0.904	Excellent

Cronbach’s Alpha ≥ 0.70 indicates acceptable reliability.

The perceived-public-health regression model explained 23.6% of variance [adjusted *R*^2^ = 0.222, *F* (7,383) = 16.889; *p* < 0.001; Table 5]. The socioeconomic-consumption composite (*B* = 0.260, 95% CI 0.145 to 0.376, *β* = 0.224, *p* < 0.001) and awareness (*B* = 0.301, 95% CI 0.208 to 0.393, *β* = 0.304, *p* < 0.001) were independently associated with stronger perceived public-health implications (Table 11). Demographic covariates were not statistically significant. The model’s explanatory value was modest.

#### 3.1.5 Community-based interventions and policy measures

Tables 13, 14 display the complete education- and occupation-stratified distributions, with response frequencies on community-based interventions and policy measures related to safe African giant snail consumption. Table 15 reports the multivariable regression and Table 5 provides the model-fit summary. Respondents generally supported the proposed food-safety measures, including environmental monitoring of harvesting areas, public education, training of local health workers, vendor regulation, safer siting of snail farms, community and school-based education, subsidised testing, protection of low-income consumers, and engagement of local markets. In the education-based policy analyses (Table 13), significant associations after Bonferroni correction remained for public education, training health workers, vendor regulation, relocating snail farms away from polluted areas, school-based education, subsidised testing, and engaging local markets. Agreement or strong agreement among secondary, tertiary, and postgraduate respondents, respectively, was 31/40, 173/209, and 110/134 for public education; 29/40, 176/209, and 113/134 for health-worker training; 31/40, 169/209, and 104/134 for vendor regulation; 30/40, 179/209, and 113/134 for relocation of farms; 28/40, 183/209, and 118/134 for school-based education; 26/40, 170/209, and 114/134 for subsidised testing; and 30/40, 167/209, and 114/134 for local-market engagement.

**Table 13:** Education-based association for community-based interventions and policy measures in Yenagoa Metropolis, Nigeria.

Questionnaire item	Educational level	Strongly disagree *n* (%)	Disagree *n* (%)	Neutral *n* (%)	Agree *n* (%)	Strongly agree *n* (%)	Total n (%)	*χ*^2^ (df)	Raw *p*	Bonferroni- adjusted p	Cramér’s V
The government should monitor the environmental quality of snail harvesting areas.	No formal education	1 (50.0%)	0 (0.0%)	0 (0.0%)	1 (50.0%)	0 (0.0%)	2 (0.5%)	27.713 (16)	0.0342	0.3421	0.133
	Primary	0 (0.0%)	2 (33.3%)	1 (16.7%)	1 (16.7%)	2 (33.3%)	6 (1.5%)				
	Secondary	1 (2.5%)	1 (2.5%)	4 (10.0%)	18 (45.0%)	16 (40.0%)	40 (10.2%)				
	Tertiary	8 (3.8%)	8 (3.8%)	29 (13.9%)	88 (42.1%)	76 (36.4%)	209 (53.5%)				
	Postgraduate	12 (9.0%)	5 (3.7%)	16 (11.9%)	54 (40.3%)	47 (35.1%)	134 (34.3%)				
Public education on food safety should include risks of trace metal exposure from snails.	No formal education	1 (50.0%)	1 (50.0%)	0 (0.0%)	0 (0.0%)	0 (0.0%)	2 (0.5%)	63.436 (16)	<0.0001	<0.0001	0.201
	Primary	0 (0.0%)	3 (50.0%)	0 (0.0%)	3 (50.0%)	0 (0.0%)	6 (1.5%)				
	Secondary	1 (2.5%)	2 (5.0%)	6 (15.0%)	14 (35.0%)	17 (42.5%)	40 (10.2%)				
	Tertiary	5 (2.4%)	6 (2.9%)	25 (12.0%)	88 (42.1%)	85 (40.7%)	209 (53.5%)				
	Postgraduate	10 (7.5%)	5 (3.7%)	9 (6.7%)	52 (38.8%)	58 (43.3%)	134 (34.3%)				
Local health workers should be trained to educate communities about snail consumption safety.	No formal education	2 (100.0%)	0 (0.0%)	0 (0.0%)	0 (0.0%)	0 (0.0%)	2 (0.5%)	95.423 (16)	<0.0001	<0.0001	0.247
	Primary	0 (0.0%)	2 (33.3%)	3 (50.0%)	1 (16.7%)	0 (0.0%)	6 (1.5%)				
	Secondary	1 (2.5%)	1 (2.5%)	9 (22.5%)	14 (35.0%)	15 (37.5%)	40 (10.2%)				
	Tertiary	4 (1.9%)	7 (3.3%)	22 (10.5%)	78 (37.3%)	98 (46.9%)	209 (53.5%)				
	Postgraduate	6 (4.5%)	2 (1.5%)	13 (9.7%)	49 (36.6%)	64 (47.8%)	134 (34.3%)				
Snail vendors should be regulated for hygiene and environmental safety practices.	No formal education	2 (100.0%)	0 (0.0%)	0 (0.0%)	0 (0.0%)	0 (0.0%)	2 (0.5%)	93.200 (16)	<0.0001	<0.0001	0.244
	Primary	0 (0.0%)	4 (66.7%)	1 (16.7%)	0 (0.0%)	1 (16.7%)	6 (1.5%)				
	Secondary	1 (2.5%)	1 (2.5%)	7 (17.5%)	19 (47.5%)	12 (30.0%)	40 (10.2%)				
	Tertiary	5 (2.4%)	10 (4.8%)	25 (12.0%)	77 (36.8%)	92 (44.0%)	209 (53.5%)				
	Postgraduate	11 (8.2%)	8 (6.0%)	11 (8.2%)	51 (38.1%)	53 (39.6%)	134 (34.3%)				
There should be policies to relocate snail farms away from polluted areas.	No formal education	2 (100.0%)	0 (0.0%)	0 (0.0%)	0 (0.0%)	0 (0.0%)	2 (0.5%)	99.624 (16)	<0.0001	<0.0001	0.252
	Primary	0 (0.0%)	1 (16.7%)	4 (66.7%)	1 (16.7%)	0 (0.0%)	6 (1.5%)				
	Secondary	1 (2.5%)	1 (2.5%)	8 (20.0%)	13 (32.5%)	17 (42.5%)	40 (10.2%)				
	Tertiary	2 (1.0%)	6 (2.9%)	22 (10.5%)	83 (39.7%)	96 (45.9%)	209 (53.5%)				
	Postgraduate	8 (6.0%)	7 (5.2%)	6 (4.5%)	54 (40.3%)	59 (44.0%)	134 (34.3%)				
Community leaders should lead campaigns about safe snail handling and cooking.	No formal education	1 (50.0%)	0 (0.0%)	0 (0.0%)	1 (50.0%)	0 (0.0%)	2 (0.5%)	22.134 (16)	0.1389	1.0000	0.119
	Primary	0 (0.0%)	1 (16.7%)	1 (16.7%)	4 (66.7%)	0 (0.0%)	6 (1.5%)				
	Secondary	1 (2.5%)	2 (5.0%)	6 (15.0%)	22 (55.0%)	9 (22.5%)	40 (10.2%)				
	Tertiary	6 (2.9%)	10 (4.8%)	20 (9.6%)	99 (47.4%)	74 (35.4%)	209 (53.5%)				
	Postgraduate	6 (4.5%)	6 (4.5%)	13 (9.7%)	60 (44.8%)	49 (36.6%)	134 (34.3%)				
Schools should educate students about environmental health and food contamination risks.	No formal education	1 (50.0%)	0 (0.0%)	0 (0.0%)	1 (50.0%)	0 (0.0%)	2 (0.5%)	56.697 (16)	<0.0001	<0.0001	0.190
	Primary	0 (0.0%)	1 (16.7%)	3 (50.0%)	2 (33.3%)	0 (0.0%)	6 (1.5%)				
	Secondary	2 (5.0%)	0 (0.0%)	10 (25.0%)	16 (40.0%)	12 (30.0%)	40 (10.2%)				
	Tertiary	5 (2.4%)	3 (1.4%)	18 (8.6%)	82 (39.2%)	101 (48.3%)	209 (53.5%)				
	Postgraduate	8 (6.0%)	3 (2.2%)	5 (3.7%)	45 (33.6%)	73 (54.5%)	134 (34.3%)				
There should be free or subsidized testing for heavy metals in local food items like snails.	No formal education	1 (50.0%)	0 (0.0%)	0 (0.0%)	1 (50.0%)	0 (0.0%)	2 (0.5%)	38.239 (16)	0.0014	0.0140	0.156
	Primary	0 (0.0%)	1 (16.7%)	2 (33.3%)	2 (33.3%)	1 (16.7%)	6 (1.5%)				
	Secondary	1 (2.5%)	1 (2.5%)	12 (30.0%)	13 (32.5%)	13 (32.5%)	40 (10.2%)				
	Tertiary	4 (1.9%)	7 (3.3%)	28 (13.4%)	81 (38.8%)	89 (42.6%)	209 (53.5%)				
	Postgraduate	6 (4.5%)	5 (3.7%)	9 (6.7%)	58 (43.3%)	56 (41.8%)	134 (34.3%)				
Policies should protect low-income groups from unsafe food practices.	No formal education	1 (50.0%)	0 (0.0%)	0 (0.0%)	0 (0.0%)	1 (50.0%)	2 (0.5%)	27.893 (16)	0.0326	0.3256	0.134
	Primary	0 (0.0%)	1 (16.7%)	2 (33.3%)	2 (33.3%)	1 (16.7%)	6 (1.5%)				
	Secondary	1 (2.5%)	0 (0.0%)	7 (17.5%)	19 (47.5%)	13 (32.5%)	40 (10.2%)				
	Tertiary	5 (2.4%)	5 (2.4%)	26 (12.4%)	97 (46.4%)	76 (36.4%)	209 (53.5%)				
	Postgraduate	7 (5.2%)	4 (3.0%)	14 (10.4%)	52 (38.8%)	57 (42.5%)	134 (34.3%)				
Engaging local markets in food safety regulation can reduce snail-related health risks.	No formal education	1 (50.0%)	0 (0.0%)	0 (0.0%)	1 (50.0%)	0 (0.0%)	2 (0.5%)	49.862 (16)	<0.0001	0.0002	0.179
	Primary	0 (0.0%)	2 (33.3%)	1 (16.7%)	3 (50.0%)	0 (0.0%)	6 (1.5%)				
	Secondary	2 (5.0%)	4 (10.0%)	4 (10.0%)	19 (47.5%)	11 (27.5%)	40 (10.2%)				
	Tertiary	8 (3.8%)	2 (1.0%)	32 (15.3%)	87 (41.6%)	80 (38.3%)	209 (53.5%)				
	Postgraduate	7 (5.2%)	3 (2.2%)	10 (7.5%)	56 (41.8%)	58 (43.3%)	134 (34.3%)				

Each response entry is reported as *n* (%); Values are expressed as frequency (n) and percentage (%); Values reported in the format n/N (%) in text indicate the number of respondents with the specified response (n), the total respondents in the relevant subgroup (N), and the corresponding percentage calculated as n divided by N multiplied by 100; Pearson *χ*^2^ with degrees of freedom, raw p-values, Bonferroni-adjusted p-values, and Cramér’s V are reported at item level. Bonferroni adjustment controls family-wise *α* = 0.05 across the 10 tests in each demographic-by-domain family.

**Table 14:** Occupation-based association for community-based interventions and policy measures in Yenagoa Metropolis, Nigeria.

Questionnaire item	Occupation	Strongly disagree *n* (%)	Disagree *n* (%)	Neutral *n* (%)	Agree *n* (%)	Strongly agree *n* (%)	Total *n* (%)	*χ*^2^ (df)	Raw *p*	Bonferroni- adjusted *p*	Cramér’s V
The government should monitor the environmental quality of snail harvesting areas.	Student	15 (7.2)	6 (2.9)	30 (14.4)	80 (38.5)	77 (37.0)	208 (53.2)	18.109 (20)	0.5802	1.0000	0.108
	Trader	1 (3.7)	3 (11.1)	4 (14.8)	12 (44.4)	7 (25.9)	27 (6.9)				
	Civil servant	5 (4.6)	5 (4.6)	12 (11.0)	45 (41.3)	42 (38.5)	109 (27.9)				
	Artisan	0 (0.0)	1 (5.0)	2 (10.0)	9 (45.0)	8 (40.0)	20 (5.1)				
	Unemployed	1 (5.6)	1 (5.6)	2 (11.1)	12 (66.7)	2 (11.1)	18 (4.6)				
	Others	0 (0.0)	0 (0.0)	0 (0.0)	4 (44.4)	5 (55.6)	9 (2.3)				
Public education on food safety should include risks of trace metal exposure from snails.	Student	13 (6.2)	9 (4.3)	27 (13.0)	76 (36.5)	83 (39.9)	208 (53.2)	37.641 (20)	0.0098	0.0979	0.155
	Trader	1 (3.7)	5 (18.5)	1 (3.7)	9 (33.3)	11 (40.7)	27 (6.9)				
	Civil servant	3 (2.8)	1 (0.9)	5 (4.6)	47 (43.1)	53 (48.6)	109 (27.9)				
	Artisan	0 (0.0)	1 (5.0)	4 (20.0)	9 (45.0)	6 (30.0)	20 (5.1)				
	Unemployed	0 (0.0)	1 (5.6)	2 (11.1)	12 (66.7)	3 (16.7)	18 (4.6)				
	Others	0 (0.0)	0 (0.0)	1 (11.1)	4 (44.4)	4 (44.4)	9 (2.3)				
Local health workers should be trained to educate communities about snail consumption safety.	Student	8 (3.8)	8 (3.8)	30 (14.4)	72 (34.6)	90 (43.3)	208 (53.2)	43.059 (20)	0.0020	0.0201	0.166
	Trader	1 (3.7)	2 (7.4)	5 (18.5)	8 (29.6)	11 (40.7)	27 (6.9)				
	Civil servant	2 (1.8)	1 (0.9)	3 (2.8)	39 (35.8)	64 (58.7)	109 (27.9)				
	Artisan	0 (0.0)	1 (5.0)	7 (35.0)	10 (50.0)	2 (10.0)	20 (5.1)				
	Unemployed	2 (11.1)	0 (0.0)	2 (11.1)	8 (44.4)	6 (33.3)	18 (4.6)				
	Others	0 (0.0)	0 (0.0)	0 (0.0)	5 (55.6)	4 (44.4)	9 (2.3)				
Snail vendors should be regulated for hygiene and environmental safety practices.	Student	11 (5.3)	12 (5.8)	24 (11.5)	71 (34.1)	90 (43.3)	208 (53.2)	32.449 (20)	0.0387	0.3874	0.144
	Trader	1 (3.7)	4 (14.8)	3 (11.1)	12 (44.4)	7 (25.9)	27 (6.9)				
	Civil servant	4 (3.7)	5 (4.6)	8 (7.3)	44 (40.4)	48 (44.0)	109 (27.9)				
	Artisan	0 (0.0)	2 (10.0)	7 (35.0)	8 (40.0)	3 (15.0)	20 (5.1)				
	Unemployed	3 (16.7)	0 (0.0)	1 (5.6)	8 (44.4)	6 (33.3)	18 (4.6)				
	Others	0 (0.0)	0 (0.0)	1 (11.1)	4 (44.4)	4 (44.4)	9 (2.3)				
There should be policies to relocate snail farms away from polluted areas.	Student	10 (4.8)	10 (4.8)	24 (11.5)	75 (36.1)	89 (42.8)	208 (53.2)	34.037 (20)	0.0259	0.2588	0.148
	Trader	1 (3.7)	2 (7.4)	8 (29.6)	8 (29.6)	8 (29.6)	27 (6.9)				
	Civil servant	1 (0.9)	2 (1.8)	4 (3.7)	48 (44.0)	54 (49.5)	109 (27.9)				
	Artisan	0 (0.0)	1 (5.0)	4 (20.0)	9 (45.0)	6 (30.0)	20 (5.1)				
	Unemployed	1 (5.6)	0 (0.0)	0 (0.0)	8 (44.4)	9 (50.0)	18 (4.6)				
	Others	0 (0.0)	0 (0.0)	0 (0.0)	3 (33.3)	6 (66.7)	9 (2.3)				
Community leaders should lead campaigns about safe snail handling and cooking.	Student	10 (4.8)	9 (4.3)	27 (13.0)	88 (42.3)	74 (35.6)	208 (53.2)	22.980 (20)	0.2898	1.0000	0.121
	Trader	1 (3.7)	2 (7.4)	3 (11.1)	15 (55.6)	6 (22.2)	27 (6.9)				
	Civil servant	2 (1.8)	5 (4.6)	6 (5.5)	55 (50.5)	41 (37.6)	109 (27.9)				
	Artisan	0 (0.0)	1 (5.0)	4 (20.0)	13 (65.0)	2 (10.0)	20 (5.1)				
	Unemployed	1 (5.6)	2 (11.1)	0 (0.0)	10 (55.6)	5 (27.8)	18 (4.6)				
	Others	0 (0.0)	0 (0.0)	0 (0.0)	5 (55.6)	4 (44.4)	9 (2.3)				
Schools should educate students about environmental health and food contamination risks.	Student	11 (5.3)	5 (2.4)	23 (11.1)	68 (32.7)	101 (48.6)	208 (53.2)	51.434 (20)	0.0001	0.0014	0.181
	Trader	2 (7.4)	0 (0.0)	8 (29.6)	11 (40.7)	6 (22.2)	27 (6.9)				
	Civil servant	2 (1.8)	0 (0.0)	1 (0.9)	44 (40.4)	62 (56.9)	109 (27.9)				
	Artisan	0 (0.0)	1 (5.0)	4 (20.0)	12 (60.0)	3 (15.0)	20 (5.1)				
	Unemployed	1 (5.6)	1 (5.6)	0 (0.0)	6 (33.3)	10 (55.6)	18 (4.6)				
	Others	0 (0.0)	0 (0.0)	0 (0.0)	5 (55.6)	4 (44.4)	9 (2.3)				
There should be free or subsidized testing for heavy metals in local food items like snails.	Student	9 (4.3)	10 (4.8)	29 (13.9)	70 (33.7)	90 (43.3)	208 (53.2)	35.660 (20)	0.0169	0.1686	0.151
	Trader	1 (3.7)	2 (7.4)	8 (29.6)	8 (29.6)	8 (29.6)	27 (6.9)				
	Civil servant	1 (0.9)	1 (0.9)	5 (4.6)	55 (50.5)	47 (43.1)	109 (27.9)				
	Artisan	0 (0.0)	1 (5.0)	6 (30.0)	9 (45.0)	4 (20.0)	20 (5.1)				
	Unemployed	1 (5.6)	0 (0.0)	2 (11.1)	9 (50.0)	6 (33.3)	18 (4.6)				
	Others	0 (0.0)	0 (0.0)	1 (11.1)	4 (44.4)	4 (44.4)	9 (2.3)				
Policies should protect low-income groups from unsafe food practices.	Student	11 (5.3)	7 (3.4)	33 (15.9)	84 (40.4)	73 (35.1)	208 (53.2)	31.533 (20)	0.0485	0.4854	0.142
	Trader	1 (3.7)	0 (0.0)	8 (29.6)	13 (48.1)	5 (18.5)	27 (6.9)				
	Civil servant	2 (1.8)	2 (1.8)	4 (3.7)	49 (45.0)	52 (47.7)	109 (27.9)				
	Artisan	0 (0.0)	1 (5.0)	2 (10.0)	11 (55.0)	6 (30.0)	20 (5.1)				
	Unemployed	0 (0.0)	0 (0.0)	2 (11.1)	9 (50.0)	7 (38.9)	18 (4.6)				
	Others	0 (0.0)	0 (0.0)	0 (0.0)	4 (44.4)	5 (55.6)	9 (2.3)				
Engaging local markets in food safety regulation can reduce snail-related health risks.	Student	12 (5.8)	5 (2.4)	33 (15.9)	78 (37.5)	80 (38.5)	208 (53.2)	48.881 (20)	0.0003	0.0032	0.177
	Trader	2 (7.4)	3 (11.1)	5 (18.5)	14 (51.9)	3 (11.1)	27 (6.9)				
	Civil servant	3 (2.8)	0 (0.0)	6 (5.5)	51 (46.8)	49 (45.0)	109 (27.9)				
	Artisan	0 (0.0)	3 (15.0)	1 (5.0)	11 (55.0)	5 (25.0)	20 (5.1)				
	Unemployed	0 (0.0)	0 (0.0)	2 (11.1)	10 (55.6)	6 (33.3)	18 (4.6)				
	Others	1 (11.1)	0 (0.0)	0 (0.0)	2 (22.2)	6 (66.7)	9 (2.3)				

Each response entry is reported as *n* (%); Values are expressed as frequency (n) and percentage (%); Values reported in the format n/N (%) in text indicate the number of respondents with the specified response (n), the total respondents in the relevant subgroup (N), and the corresponding percentage calculated as n divided by N multiplied by 100; Pearson *χ*^2^ with degrees of freedom, raw *p*-values, Bonferroni-adjusted p-values, and Cramér’s V are reported at item level. Bonferroni adjustment controls family-wise *α* = 0.05 across the 10 tests in each demographic-by-domain family.

**Table 15:** Multiple linear regression analysis of community-based interventions and policy measures in Yenagoa Metropolis, Nigeria.

Predictor	B	SE	β	t	P	95% CI for B	Tolerance	VIF
Constant	1.728	0.406	—	4.256	<0.001	0.930 to 2.526	—	—
Age	0.222	0.108	0.133	2.059	0.040	0.010 to 0.434	0.506	1.976
Sex	0.050	0.084	0.030	0.601	0.548	−0.114 to 0.215	0.858	1.166
Marital status	−0.109	0.080	−0.081	−1.372	0.171	−0.266 to 0.047	0.597	1.676
Educational level	0.156	0.057	0.134	2.745	0.006	0.045 to 0.267	0.878	1.139
Occupation	0.021	0.036	0.034	0.583	0.560	−0.050 to 0.093	0.613	1.632
Socioeconomic-consumption composite	−0.099	0.061	−0.086	−1.616	0.107	−0.219 to 0.022	0.746	1.340
Awareness composite	0.021	0.050	0.021	0.412	0.681	−0.078 to 0.120	0.795	1.258
Perceived public-health composite	0.421	0.052	0.425	8.114	<0.001	0.319 to 0.523	0.764	1.309

B, Unstandardized coefficient; SE, Standard error; *β*, Standardized coefficient; CI, 95% confidence interval; VIF, variance inflation factor. Coefficients are cross-sectional associations, not causal effects.

After Bonferroni correction across the 10 occupation-based policy tests, significant associations remained for training local health workers (adjusted *p* = 0.0201), school-based environmental-health education (adjusted *p* = 0.0014), and engagement of local markets in food-safety regulation (adjusted *p* = 0.0032). Table 14 shows that agreement or strong agreement with health-worker training was recorded by 103/109 (94.5%) civil servants, 162/208 (77.9%) students, 19/27 (70.4%) traders, and 12/20 (60.0%) artisans. For school-based education, agreement or strong agreement was recorded by 106/109 (97.2%) civil servants, 169/208 (81.2%) students, and 17/27 (63.0%) traders. For local-market engagement, the corresponding frequencies were 100/109 (91.7%), 158/208 (76.0%), and 17/27 (63.0%).

The policy-support regression model explained 19.9% of variance (adjusted *R*^2^ = 0.182), *F* (8,382) = 11.877, *p* < 0.001 (Table 5). Age (B = 0.222, 95% CI 0.010 to 0.434, *β* = 0.133, *p* = 0.040), education (B = 0.156, 95% CI 0.045 to 0.267, *β* = 0.134, *p* = 0.006), and perceived public-health implications (B = 0.421, 95% CI 0.319 to 0.523, *β* = 0.425, *p* < 0.001) were independently associated with higher policy-support scores (Table 15). Sex, marital status, occupation, the socioeconomic-consumption composite, and awareness were not statistically significant. These associations describe differences in support within the observed sample.

#### 3.1.6 Reliability analysis of questionnaire constructs

In the main analytical sample, internal consistency was good to excellent across the four prespecified domains (Table 12): socioeconomic factors related to snail consumption *α* = 0.806; awareness, perceptions, and coping strategies concerning snail safety *α* = 0.849; perceived public-health implications and vulnerability *α* = 0.855; community-based interventions and policy measures *α* = 0.948; and overall 40-item *α* = 0.904. These are main-study coefficients and are distinct from the lower 0.72–0.88 range obtained during the 20-person pretest.

#### 3.1.7 Secondary comparison of the four composite domain scores

The mean composite scores were 2.680 (SD 0.722) for socioeconomic factors related to snail consumption, 3.016 (SD 0.851) for awareness, perceptions, and coping strategies concerning snail safety, 3.109 (SD 0.841) for perceived public-health implications and vulnerability, and 4.096 (SD 0.833) for community-based interventions and policy measures. Because Mauchly’s test was significant, the Greenhouse–Geisser-corrected within-subject effect was used, *F* (2.795,1089.977) = 306.054, *p* < 0.001, partial η^2^ = 0.440. Bonferroni-adjusted pairwise comparisons indicated that Domain 2 and Domain 3 did not differ significantly (adjusted *p* = 0.294), whereas all other domain-pair differences were statistically significant. These comparisons are descriptive because the four domains represent different constructs despite sharing a 1–5 metric.

### 3.2 Discussion

#### 3.2.1 Socioeconomic and demographic characteristics of respondents

As shown in Table 1, the achieved sample was concentrated among students and persons with higher education and included more women than men. The pattern is consistent with the venue-based component of recruitment and reinforces the study’s limitation regarding population generalisability. It also means that comparisons with population-based studies should focus on patterns of association rather than direct prevalence equivalence.

The sample profile differs from producer-focused snail studies in Nigeria, which commonly recruit farmers and therefore reflect different occupational and household characteristics. Studies from Oyo and Edo States have described snail production largely among economically active farming households (28, 29). The contrast with the present consumer-focused sample is therefore more probably related to differences in target populations and recruitment settings than to a contradiction in the literature. This distinction is important because producer characteristics cannot be assumed to represent the demographic profile of urban consumers.

Figure 1 indicates that respondents perceived both environmental contamination and hygiene-related pathways as relevant to snail safety. This perception is biologically plausible because laboratory studies have shown that edible land snails can accumulate trace metals and that concentrations vary by species and harvesting location (13–15). Microbiological investigations have also reported bacterial contamination in snail samples (30). The values are seen as evidence of perceived contamination pathways.

Accordingly, the environmental and hygiene concerns represented in Figure 1 are best seen as consumer risk perceptions that are consistent with known contamination pathways. This helps preserves the usefulness of the perception data for food-safety communication while avoiding an unsupported inference from perceived contamination to measured exposure. It also provides a clearer basis for recommending risk communication and source traceability.

#### 3.2.2 Socioeconomic factors related to snail consumption

Tables 2, 3 show that demographic differences in the socioeconomic-consumption items were selective rather than uniform. After correction for multiple testing, differences persisted for a subset of beliefs concerning nutritional benefits, affordability, cultural or household context, gender-related preference, and socioeconomic patterns. The accompanying Cramér’s V values indicate modest group separation, so the findings seen as patterned response differences rather than strong demographic determinants.

These findings complement Nigerian studies describing the economic and food-security roles of snail production (28, 29), but the present study addresses consumer perceptions rather than farm economics. The study do not establish that income, employment, education, or household characteristics cause the reported consumption patterns. Instead, the results indicate that socioeconomic and cultural beliefs are distributed differently across some respondent groups. This explanation is more consistent with the observational design and with the modest effect sizes shown in Tables 2, 3.

Table 4 indicates that education and marital status retained independent associations with the composite outcome after adjustment. The inverse association with education should not be seen as evidence that education reduces snail consumption or that food-safety awareness mediates the relationship. The present regression therefore identifies independent statistical relationships within the sample rather than behavioural pathways. The item-level patterns in Tables 2, 3 also show that cultural and family-related perceptions remain relevant to how respondents describe snail consumption. These findings are consistent with the broader social and household context reported in Nigerian snail-production research. Cultural preference may operate alongside affordability, availability, and learned food practices rather than as an isolated explanation. The study therefore support a multidimensional understanding of consumption-related perceptions rather.

Table 5 further shows that the demographic model has limited explanatory power. This supports a restrained understanding in which measured demographic characteristics explain part of the variation in the socioeconomic-consumption domain. Behavioural, market, cultural, and environmental factors that were not included in the model are therefore likely to contribute to the remaining variation. The model is consequently more useful for identifying correlates than for predicting individual consumption-related responses.

#### 3.2.3 Awareness, perceptions, and coping strategies concerning snail safety

Tables 7, 8 show uneven awareness and protective behaviour. After multiplicity correction, occupation remained associated with selected items concerning pollution awareness, source checking, and vendor hygiene, whereas education-based differences did not persist. This pattern suggests that occupational context may correspond with some information and purchasing practices in this sample. It also shows that statistical differences reflect shifts in response distributions rather than uniformly high or low awareness within any single occupation.

The concern expressed by respondents is consistent with laboratory evidence that edible land snails can accumulate trace metals from their environment (13, 14). However, awareness of a potential contamination pathway and actual protective behaviour are distinct, which is why the source-checking and vendor-hygiene findings in Tables 7 and 8 are important for risk communication. Knowledge alone may not translate into protective purchasing if consumers lack reliable information about harvesting location, vendor practices, or product traceability. The observed awareness-practice gap therefore has practical relevance.

Visual inspection or vendor appearance cannot show whether trace metals are present in food. Consequently, the findings support communication around traceable sourcing and environmental monitoring without implying that respondents can identify chemical contamination through appearance alone (14, 15). Consumer education should therefore distinguish visible hygiene indicators from contaminants that require laboratory measurement. This distinction can strengthen food-safety messaging while avoiding false reassurance based on appearance.

The multivariable findings in Table 6, presented alongside the model summary in Table 5, indicate that the socioeconomic-consumption composite was related to awareness while the demographic covariates did not show independent associations. The model’s limited explanatory performance suggests that important influences on awareness were not captured by the variables included in the analysis. Such influences may include access to food-safety information, prior experience, trust in institutions, or characteristics of local food markets, although these mechanisms were not directly tested. The regression should therefore be seen as a partial explanatory model.

#### 3.2.4 Perceived public-health implications and vulnerability

Tables 9, 10 show that perceived vulnerability differed across demographic groups for only a limited subset of items after correction, particularly perceptions concerning low-income families and children. These results describe beliefs about vulnerability rather than measured exposure or disease. The distinction is important because demographic differences in perceived risk cannot be seen that the named groups actually experienced higher contaminant doses.

Laboratory studies from southern Nigeria have measured metals in edible snails and, in some settings, estimated dietary health risks (14–16, 31). Those studies establish the plausibility of food-safety concern in specific sampled locations, whereas the present questionnaire data establish only how respondents perceived potential public-health implications. The two forms of evidence are complementary.

Table 11 shows that the questionnaire domains were associated within the multivariable model, but such relationships remain perceptual and behavioural. Environmental contamination is location-, species-, and exposure-specific, and direct chemical analysis is necessary before conclusions can be made about actual trace-metal concentrations or dietary exposure (13–15). The observed relationships may nevertheless be useful for understanding how consumption-related beliefs and awareness correspond with perceived public-health implications.

The model-fit summary in Table 5 indicates that the perceived-public-health model performed better than the earlier domain models but still left substantial variation unexplained. The findings therefore support relationships among the questionnaire constructs. The remaining unexplained variance is consistent with the likelihood that risk perception is shaped by additional social, experiential, informational, and environmental influences.

#### 3.2.5 Community-based interventions and policy measures

Tables 13, 14 show broad support for the proposed food-safety measures, with demographic differences persisting for selected items after multiplicity correction. Because support was generally high, the statistically significant crosstabulations should be understood as differences in the distribution or intensity of endorsement These findings are therefore most useful for identifying areas of consensus and potential differences in emphasis across demographic groups.

The multivariable findings in Table 15, together with Table 5, indicate that perceived public-health implications were the most prominent correlate of policy support, while age and education showed smaller independent relationships. Respondent support can identify communication and engagement priorities, but it does not establish whether an intervention is feasible, affordable, enforceable, or effective at reducing contamination. Policy acceptance should therefore be regarded as one component of intervention design. Feasibility and impact would require separate implementation, economic, environmental, and outcome evaluation.

The effect-size information in Tables 13, 14 also tempers understanding because the corrected associations represent modest demographic separation rather than sharply divided policy preferences. Policy development should therefore combine public acceptability evidence with environmental monitoring, implementation assessment, economic evaluation, and measured food-safety outcomes. The broad endorsement observed across many strata suggests that risk-communication and monitoring initiatives may have a favourable basis for public engagement, but this inference concerns acceptability rather than effectiveness.

#### 3.2.6 Reliability of the measurement instrument

Table 12 indicates good to excellent internal consistency across the prespecified questionnaire domains. This indicates that the instrument reliably measures socioeconomic factors, awareness, public health perceptions, and policy measures. This finding aligns with Izah (32) on consumption patterns in Yenagoa metropolis. High reliability values are consistent with established methodological standards (33), confirming the robustness of the instrument. The strong reliability of the policy construct further indicates consistency in respondents’ views on intervention strategies. This supports the suitability of the instrument for similar public health and environmental studies in comparable perspective.

#### 3.2.7 Secondary comparison of the four composite domain scores

The secondary within-respondent comparison in Table 16 shows that the community-based interventions and policy-measures domain was more strongly endorsed on the common five-point metric, while the awareness and perceived-public-health domains were comparatively similar. Because the domains represent different constructs, these pairwise comparisons are best seen as descriptive contrasts in response intensity rather than a ranking of conceptual importance. The result nonetheless indicates that respondents expressed particularly strong endorsement of intervention-oriented statements relative to the other domains.

**Table 16:** Bonferroni-adjusted pairwise comparisons of mean composite domain scores.

Comparison	Mean difference	SE	Bonferroni *p*	95% CI
Socioeconomic factors related to snail consumption vs. awareness, perceptions, and coping strategies concerning snail safety	−0.336	0.046	<0.001	−0.458 to −0.214
Socioeconomic factors related to snail consumption vs. perceived public-health implications and vulnerability	−0.429	0.045	<0.001	−0.547 to −0.310
Socioeconomic factors related to snail consumption vs. community-based interventions and policy measures	−1.416	0.054	<0.001	−1.560 to −1.272
Awareness, perceptions, and coping strategies concerning snail safety vs. perceived public-health implications and vulnerability	−0.093	0.047	0.294	−0.217 to 0.032
Awareness, perceptions, and coping strategies concerning snail safety vs. community-based interventions and policy measures	−1.080	0.055	<0.001	−1.227 to −0.933
Perceived public-health implications and vulnerability vs. community-based interventions and policy measures	−0.987	0.047	<0.001	−1.111 to −0.864

Pairwise *p*-values and confidence intervals are Bonferroni-adjusted estimated-marginal-mean comparisons from the repeated-measures GLM.

### 3.3 Study limitations

The achieved sample was dominated by students and persons with tertiary or postgraduate education, reflecting the venue-based component of recruitment and limiting generalisability to the full adult population of Yenagoa Metropolis. Also, the study also showed a significant number are students and had at least tertiary education, thus limiting generalizability of the total adult population in the metropolis. Furthermore, the study is cross-sectional, so observed associations cannot establish temporal order or causality.

## 4 Conclusion and recommendations

African giant snail consumption and related food-safety perceptions in this sample were associated with selected demographic and attitudinal characteristics, but the explanatory power of the regression models was modest. Respondents reported awareness of possible environmental contamination and expressed strong support for food-safety interventions. The study provides evidence about consumer perceptions and self-reported practices. The findings also identify areas of public support that may inform the design of future food-safety programmes. Environmental monitoring of harvesting environments, risk communication on traceable sourcing, vendor hygiene oversight, school and community education, and accessible food testing were widely supported by respondents. However, implementation decisions should be based on separate assessments of feasibility, cost, regulatory capacity, and measured contamination.

- Where environmental agencies and public-health authorities consider monitoring programmes, priority may be given to areas with documented pollution concerns.
- Health-education programmes may emphasise the difference between visible cleanliness and chemical safety, encourage source verification where practicable, and communicate that contamination risk accordingly.
- Vendor- and market-focused measures may prioritise basic hygiene standards, traceability of harvesting sources, and risk-based laboratory sampling of products.

## Data Availability

The original contributions presented in the study are included in the article/supplementary material, further inquiries can be directed to the corresponding author/s.
